# A heuristic approach to evaluate *peri* interactions *versus* intermolecular interactions in an overcrowded naphthalene

**DOI:** 10.1107/S205225251601808X

**Published:** 2017-01-01

**Authors:** Sounak Sarkar, Tayur N. Guru Row

**Affiliations:** a Solid State and Structural Chemistry Unit, Indian Institute of Science, C. V. Raman Avenue, Bangalore, Karnataka 560012, India

**Keywords:** overcrowding, *peri* interaction, charge density, aromaticity, halogen bonding, intermolecular interactions, charge spin and momentum densities

## Abstract

Octachloronaphthalene (OCN), a serious environmental pollutant, has been investigated by charge density analysis to unravel several unexplored factors responsible for steric overcrowding. The topological features of the enigmatic *peri* interactions contributing to steric overcrowding are qualified and quantified from experimental and theoretical charge-density studies. A new facet in the fundamental understanding of *peri* interactions is revealed by NCI (non-covalent interaction) analysis. The potential role of these interactions in deforming the molecular geometry and subsequent effect on aromaticity are substantiated from NICS (Nuclear Independent Chemical Shift) and QTAIM (Quantum Theory of Atoms in Molecules) calculations. The eye-catching dissimilarity in the out-of-plane twisting of OCN renders the molecule in an asymmetric geometry in the crystalline phase compared with symmetric geometry in the optimized solvated phase. This is uniquely characterized by their molecular electrostatic potential (MESP), respectively, and is explained in terms of conflict between two opposing forces – *peri* interactions, and symbiotic intermolecular Cl⋯Cl and Cl⋯π contacts.

## Abstract 2

An electron density study on sterically overcrowded octachloronaphthalene is performed using an extensive combination of high-resolution single crystal X-ray diffraction and *ab initio* single-point and periodic DFT calculations. The unusual asymmetry in the crystalline geometry as opposed to symmetric solvated phase geometry is investigated and the role of steric forces, contributed primarily by the *peri* interactions in imparting unique ‘localized double bond’ character in the naphthalene ring, is rationalized.

## 1. Introduction   

The term ‘overcrowding’ in chemistry is synonymous with the presence of non-bonded intramolecular interactions often referred to as steric hindrance. These steric factors influenced by the presence of exocyclic bonds invoke in-plane or out-of-plane deviations from the ideal geometry of aromatic molecules. Consequently, the molecules undergo geometrical manifestation to release the strain in the system which in turn modulates the π-electron delocalization. This results in an alteration in the reactivity and property of the molecule, a factor of significant interest to chemists. The synthesis of sterically congested polyaromatic hydrocarbons (PAHs) has remained a challenge due to their elusiveness often resulting in low yields. The design of convex, bowl-shaped PAHs akin to fullerenes and also as synthetic precursors for the synthesis of carbon nanotubes has been a matter of interest to chemists for decades (Wu & Siegel, 2006 ▸; Scott *et al.*, 2012 ▸). PAHs have garnered considerable attention in recent times due to their potential applications in organic electronics (Tannaci *et al.*, 2007 ▸; Pascal, 2006 ▸). In addition, highly symmetric PAHs form cavities in a supramolecular assembly with the potential to encapsulate and trap guest molecules (Downing *et al.*, 1994 ▸). Overcrowded bistricyclic aromatic enes (BAEs) show thermochromic and photochromic properties which qualify them as candidates for chiroptical molecular switches and molecular motors (Biedermann *et al.*, 2001 ▸). Overcrowding in aromatic molecules such as naphthalene, acenaphthylenes, acenaphthene and pyrene are uniquely characterized by *peri* interactions (Balasubramaniyan, 1966 ▸; Diamond *et al.*, 2014 ▸; Knight *et al.*, 2012 ▸). In the case of compounds wherein atoms or groups other than H atoms are at the *peri* positions like in naphthalene and related PAHs, the ‘ideal’ *peri* distance (*ca* 2.5 Å) between respective atoms or groups becomes shorter than the sum of their van der Waals radii. This causes the atoms or groups to crave for space leading to steric congestion in the molecule. In order to minimize these steric strains the rigid carbon framework undergoes distortion that generally leads to extended distances. The extent of distortions or the degree of strain in these systems depends upon the number, size and the nature of the functional groups present at the *peri* positions and also the type of aromatic backbone involved. Several synthetic routes have been suggested for the preparation of *peri* substituted naphthalene, acenaphthylenes and acenaphthene, and efforts have been made on exploring the nature of weak non-bonded intramolecular interactions in the *peri* substituted species (Hoefelmeyer *et al.*, 2002 ▸; Dominiak *et al.*, 2005 ▸; Kilian *et al.*, 2011 ▸; Matta *et al.*, 2005 ▸). The transannular interactions between substituents in 1,8- and 5,6-positions can either be repulsive due to steric congestion which result from the direct overlap of orbitals (lone pair–lone pair interactions for Groups 15, 16, 17) or attractive due to *n*(lone-pair) → σ*(antibonding) orbital interactions. The majority of these studies were made based on crystal structure analysis and routine gas-phase calculations. Indeed, a very limited number of experimental electron density studies involving *peri* interactions are reported in the literature (Mallinson *et al.*, 1999 ▸; Lyssenko *et al.*, 2004 ▸; Farrugia *et al.*, 2009 ▸; Hoser *et al.*, 2010 ▸). However, all such studies are devoid of detailed quantitative and qualitative discussion on the nature of these non-conventional intramolecular interactions and their influence on the molecular geometry and π-electron distribution.

**Figure 1:**
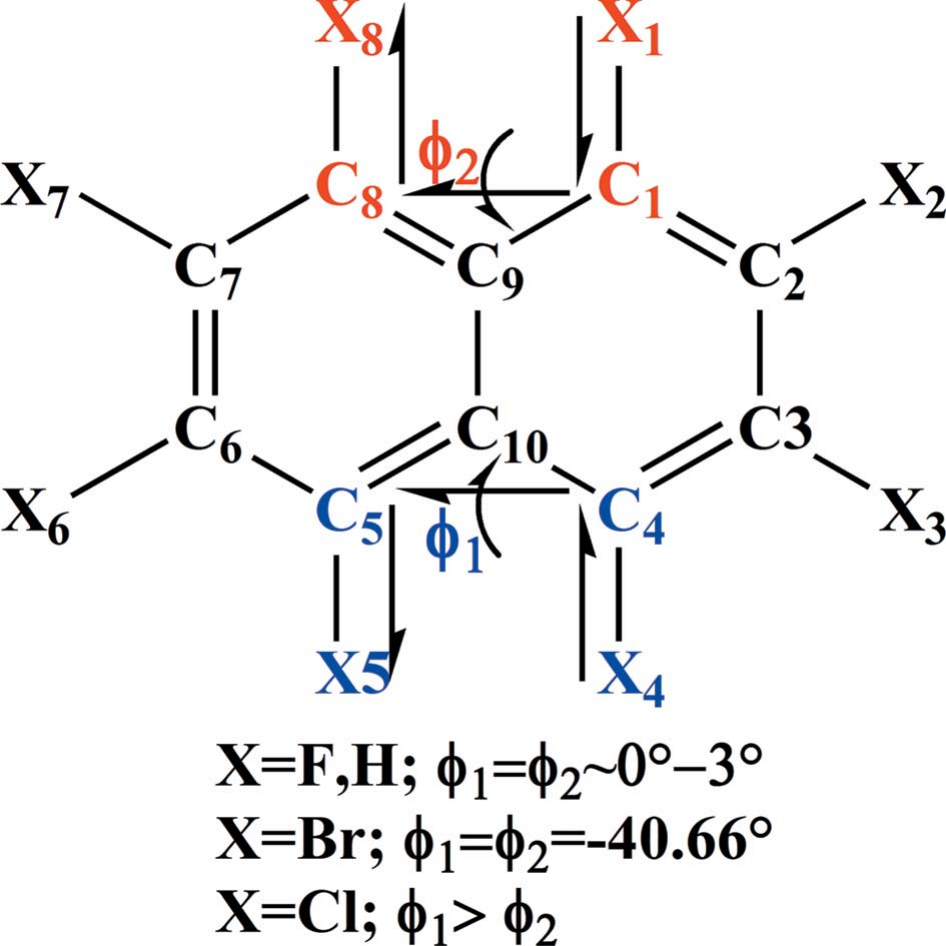
Representation of ϕ_1_ and ϕ_2_ dihedral angles in the schematic diagram of naphthalene and octahalonaphthlene.

Halogenated naphthalenes show anomalous gradation in intra- and intermolecular interactions and associated crystal packing based on the size of the halogen atom. The degree of polarization in a halogen atom and its supramolecular effects has been a topic of intense investigations towards understanding intermolecular interactions and rational design of molecular crystals (Gilday *et al.*, 2015 ▸). An analysis based on the Cambridge Structural Database (CSD; Version 5.37, November 2015) reveals an interesting trend in the crystal structures of the octahalo derivatives of naphthalene. The smaller size of fluorine (isosteric to hydrogen) has no overcrowding effect in the octafluoronaphthalene such that the molecule remains planar with a centre of symmetry passing through the centre of the molecule in the crystal structure (Ilott *et al.*, 2010 ▸). Octabromonaphthalene understandably exhibits an out-of-plane twisted conformation because of larger bromine atoms giving rise to steric effects; although it is non-planar, the twisted conformation is symmetrical making the molecule symmetrical such that it too sits on a twofold rotation axis (Brady *et al.*, 1982 ▸). However, in sharp contrast, octachloronaphthalene (hereafter referred as OCN) shows a twisted conformation which is asymmetric in nature, *i.e.* the molecular geometry is asymmetric (Fig. 1 ▸), and therefore the molecule sits on a general position in the crystal structure. These features prompted us to closely investigate the anomalous behaviour of OCN to gain insight into the overcrowding phenomenon and its effects on geometry, aromaticity and supramolecular assembly. It is noteworthy that the current study on OCN is the first X-ray electron density study on an overcrowded molecule and the results are expected to provide a fundamental understanding of the overcrowding phenomenon. In addition, the importance of this study comes from the fact that OCN, which belongs to the class of polychlorinated naphthalenes (PCNs), is a serious environmental pollutant (Falandysz, 1998 ▸). Toxicological studies of OCN based on metabolic reactivity on living cells (Campbell *et al.*, 1981 ▸) and thermal degradation of OCN (Su *et al.*, 2014 ▸) are of huge relevance in the context of environmental protection. Further, nucleophilic substitution reactions of OCN produces an α-substituted product contrary to the β-substituted product obtained in the case of octafluoronaphthalene (Brady *et al.*, 1984 ▸). To gain a clear perception on the nature of these properties/reactivities of OCN and their correlation with its structure, understanding of the electron density distribution is deemed significant.

OCN in its reported room-temperature (Herbstein, 1979 ▸) structure displays remarkably shorter distances (nearly 17% shorter than the sum of van der Waals radii) between *peri* Cl atoms on either side of the molecule. The influence of such transannular Cl⋯Cl interactions at the 1,8 and 4,6 *peri* substituted positions further triggered by the overcrowding of the other Cl atoms is suggested to possibly cause the molecule to deviate from the plane of symmetry.

In this work the nature of the *peri* interactions is analyzed and the impact of the conflict/contest between such intramolecular *peri* interactions with other intermolecular interactions on the molecular geometry as well as on the crystal packing is investigated. The multipole model derived at high-resolution X-ray data (Hansen & Coppens, 1978 ▸), along with topological analysis based on Bader’s QTAIM (Bader, 2002 ▸) approach, provide a qualitative description of bonding features and quantitative inputs to the properties exhibited in crystals. It is of interest to note that an unusual difference in the out-of-plane displacements occurs in the OCN molecule. Analysis of the packing features in terms of the nature of electron density distribution in the crystal structure both from theory and experiment provides an explanation. The degree of aromaticity lost in the molecule because of out-of-plane deformations is evaluated in terms of the ellipticity profile (Farrugia *et al.*, 2009 ▸) and Nuclear Independent Chemical Shift (NICS) studies (Chen *et al.*, 2005 ▸).

## 2. Experimental   

### 2.1. Synthesis   

The compound octachloronaphthalene (OCN) was synthesized according to the method reported in the literature (Jacobsson *et al.*, 2007 ▸).

### 2.2. Crystallization   

The purified solid was kept for crystallization in a saturated solution of benzene at low temperature (278 K). Yellow needle crystals were obtained after 2 weeks of solvent evaporation.

### 2.3. Data collection and structure refinement details   

The high-resolution charge-density data on OCN were collected on an Oxford Xcalibur (Mova) diffractometer equipped with an EOS CCD detector using Mo *K*α radiation (λ = 0.71073 Å). A crystal of dimensions 0.34 × 0.21 × 0.06 mm, was cooled to 100 K with a liquid nitrogen stream using an Oxford cobra open stream non-liquid nitrogen cooling device. The crystal-to-detector distance was fixed at 45 mm, and the scan width (Δω) was 1° per frame during the data collection. The data collection strategy was chosen in such a way to yield a high resolution X-ray data set (*d* = 0.46 Å) with high redundancy and completeness to 100%. Cell refinement, data integration and reduction were carried out using the program *CrysAlisPro* (Oxford Diffraction, 2011 ▸). Face indexing was done for the accurate numerical absorption correction. Sorting, scaling and merging of the data sets were carried out using the program *SORTAV* (Blessing, 1997 ▸). The crystal structure was solved by direct method using *SHELXS*2014 (Sheldrick, 2014 ▸) and refined based on the spherical-atom approximation (based on *F* ^2^) using *SHELXL*2014 included in the *WinGX* package suite (Farrugia, 2012 ▸).

### 2.4. Multipole modelling   

The charge density modelling and multipolar aspherical atom refinements for OCN were performed based on the Hansen and Coppens multipole formalism using *XD*2015 (Koritsanszky *et al.*, 2015 ▸). The function Σ*w*[|*F*_o_|^2^ − |*F*_c_|^2^]^2^ was minimized for all reflections with *I* > 2σ(*I*). Weights (*w*) were taken as 1/σ^2^(*F*_o_^2^), and the convergence criterion of the refinement was set to a maximal shift/e.s.d. of < 10^−10^. From the list of scattering factor wavefunctions available in the *XD* package, the basis functions and single-ζ values were taken from the databank file of Su−Coppens−Macchi (Su & Coppens, 1998 ▸). The scale factor was refined against the whole resolution range of diffraction data in the first refinement step. The scatterplot depicting the variation of *F*_obs_ with *F*_calc_ and variation of *F* ^2^_obs_/*F* ^2^_calc_ with (sinθ)/λ (supporting information, Fig. S1) is indicative of the good quality of the data after scaling. The positional and anisotropic displacement parameters of all the atoms were refined against the whole resolution of data. The multipolar populations were constrained to obtain a reasonable data-to-parameter ratio. Further scale, positional and anisotropic displacement parameters, *P*_val_, *P*_lm_, κ, and κ′, on all atoms were refined in a stepwise manner, until the convergence criterion was reached. Separate κ and κ′ parameters were used to define different atom types based on their chemical environments. The multipole expansion was extended to hexadecapoles in the case of the chlorine atoms (*l* = 4) and were truncated at the octupole level for carbon atoms (*l* = 3). The residual electron density peaks in the final model are −0.44 and 0.47 e Å^−3^ with an r.m.s. value of 0.09 e Å^−3^ for minimum and maximum values, respectively, at full resolution (1.08 Å^−1^, supporting information, Fig. S2). The fractal dimension plot (Meindl & Henn, 2008 ▸) which provides the overall distribution of residual electron density in the unit cell is symmetric in nature and parabolic in shape (sin θ/λ ≤ 0.8 Å^−1^) (supporting information, Fig. S3). The quantitative analysis of the electron-density topology and related properties was performed using the *XDPROP* (Volkov *et al.*, 2000 ▸) module of the *XD* software suite. The relevant crystallographic and refinement details are listed in Table 1 ▸ and the multipole population parameters are provided in the supporting information (Tables S1–S5).

## 3. Results and discussion   

The outcomes from the high-resolution X-ray diffraction data at 100 K, experimental charge-density modeling and gas-phase DFT calculations are organized into four sections. In §3.1, the crystal structure is discussed in detail. §3.2 describes the nature of intramolecular *peri* interactions, while in §3.3 the effect of overcrowding on π-electron distribution and consequently on aromaticity is determined. §3.4 focuses on the investigation of the observed asymmetric twisted conformation (differences in dihedral angles) in the crystal and also establishes the importance of interplay between intramolecular steric forces and intermolecular interactions in directing the conformation as well as crystal packing.

### 3.1. Crystal structure at 100 K   

**Figure 2:**
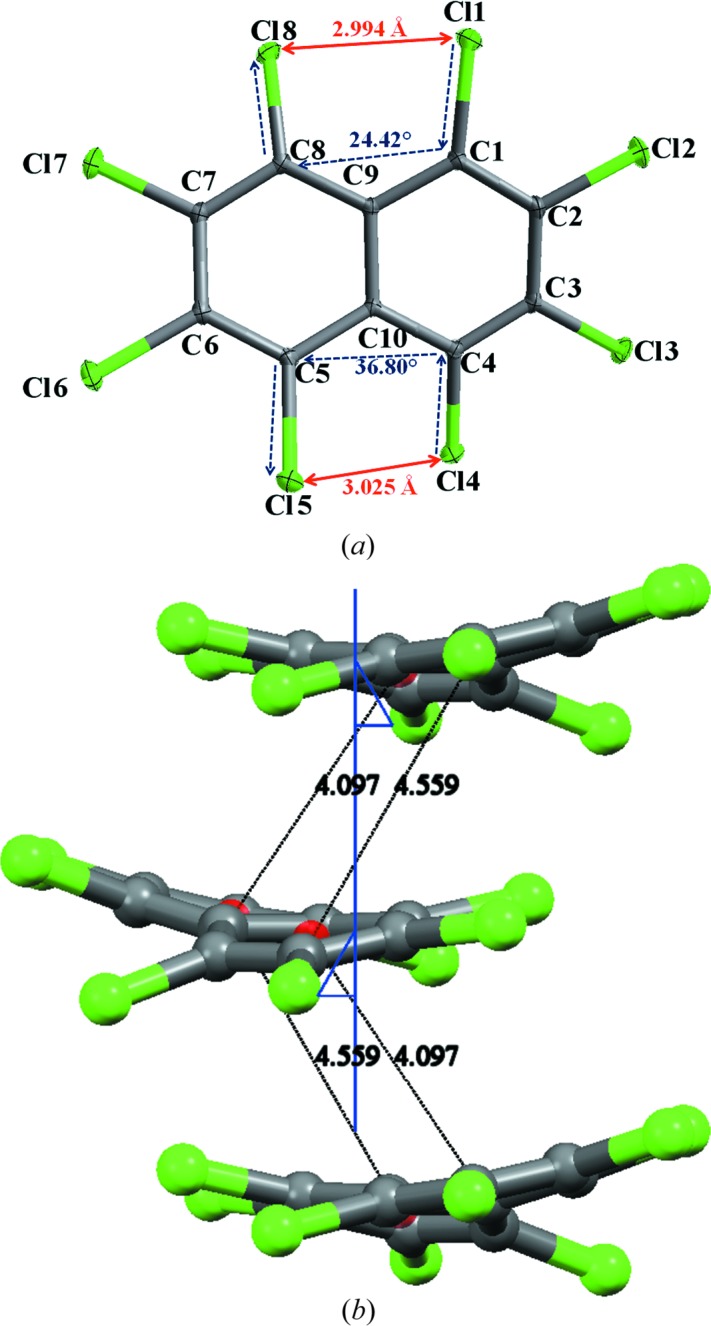
(*a*) *ORTEP* representation of the structure of OCN at 100 K. Atom ellipsoids are shown at the 50% probability level. (*b*) Molecular packing diagram along the 2_1_ screw axes parallel to the *b* axis.

OCN crystallizes in the monoclinic space group *P*2_1_/*n*. The salient features of the crystal structure are the presence of two distinct short intramolecular *peri* Cl⋯Cl interactions (1,8 = 2.994 Å and 4,6 = 3.025 Å) and two different dihedral values of angles ϕ_1_ and ϕ_2_ at the *peri* substituted positions ϕ_1_(Cl4—C4—C6—Cl5 = 36.80°) and ϕ_2_(Cl1—C1—C9—Cl8 = 24.42°) (Fig. 2 ▸ *a*). This confirms the asymmetry between the upper and lower halves of the molecule. In the intermolecular space, the molecules aggregate along the 2_1_ screw axes parallel to the *b* axis in parallel-displaced geometries (Fig. 2 ▸ *b*). However, due to the twisted conformation of OCN, π–π stacking is inefficient as vertical separation between centres of the corresponding fused benzene rings (in the individual naphthalene moiety) are significantly longer (4.097 and 4.559 Å, respectively) than the optimum intermolecular distance of ∼ 3.75 Å between two benzene rings in a parallel displaced configuration generally observed in crystal structures (Sinnokrot *et al.*, 2002 ▸). The presence of Cl atoms at all positions of overcrowded naphthalene molecule gives rise to various types of interesting intermolecular Cl⋯Cl interactions (supporting information, Figs. S4 and S5).

A Cambridge Structural Database (CSD) analysis was carried out to estimate the abundance of intramolecular Cl⋯Cl interactions in molecular crystals. The search was done on the basis of the Cl⋯Cl contact distance being restricted to the sum of the van der Waals radii (Batsanov, 2001 ▸) and the corresponding intramolecular contacts are separated by at least four covalent bonds. The search resulted in 421 hits (supporting information, Fig. S6), out of which 35 have intramolecular *peri* Cl⋯Cl interactions. The distances found for the *peri* Cl⋯Cl interactions in OCN are in agreement with the average value of 3.000 ± 0.025 Å derived from the histogram of the number of hits *versus* distance (supporting information, Fig. S7).

### 3.2. Nature of intramolecular Cl⋯Cl *peri* interactions   

In order to unequivocally establish the nature of *peri* interactions and their possible influence on the deviation from planarity and unusual molecular geometry, Bader’s topological and RDG-based NCI analysis have been performed and are outlined below.

#### 3.2.1. Topological analysis   

**Figure 3:**
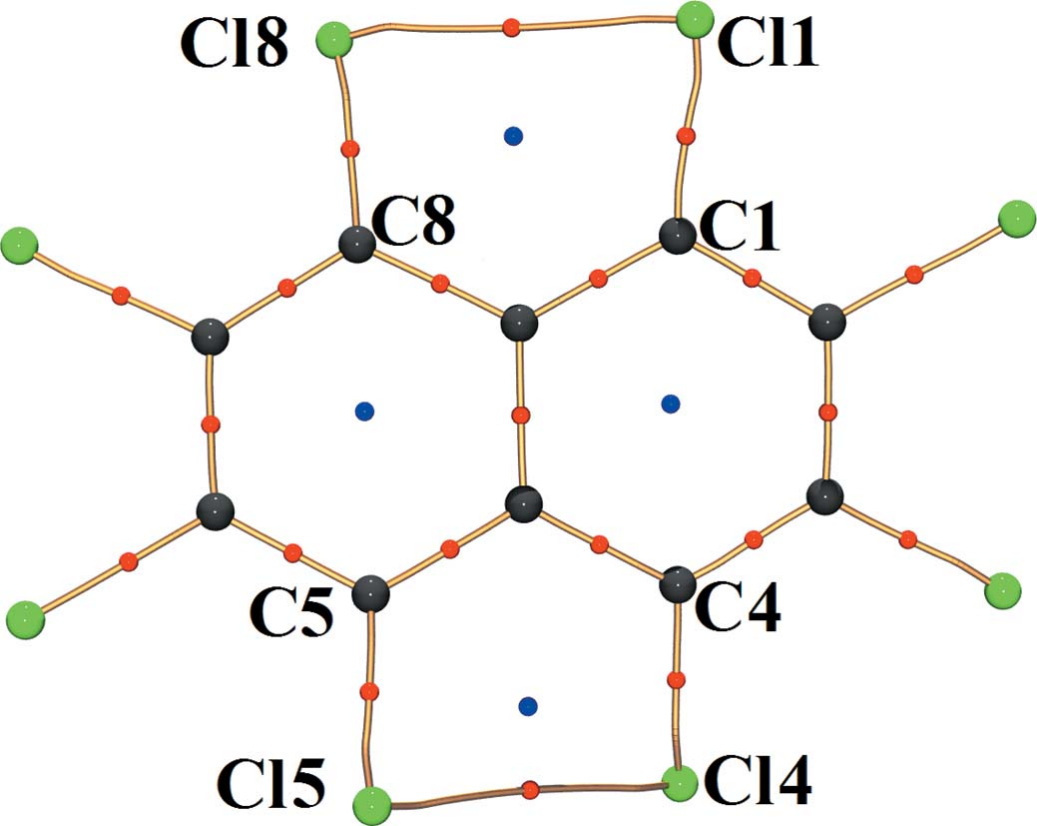
Molecular graph depicting experimental bond paths along with critical points of *peri* Cl⋯Cl interactions. The blue dots represent the (3,+1) ring critical point (RCP) while the red dots represent the (3,−1) bond critical points (BCP). The curved segments signifies the bond paths between atoms.

Topological analysis based on QTAIM theory (Bader, 2002 ▸) locates (3,−1) bond critical points (BCPs) between *peri* substituted Cl atoms (Fig. 3 ▸). The topological descriptors such as *R_ij_* (interaction distance; Å), ρ (electron density; e Å^−3^), ∇^2^ρ (second derivative of electron density; e Å^−5^), ∊ (ellipticity = (λ_1_/λ_2_) −1), and *G* (kinetic energy density; kJ mol^−1^ bohr^−3^) and *V* (potential energy density; kJ mol^−1^ bohr^−3^) for the *peri* Cl⋯Cl interactions evaluated at the BCPs of these interaction regions are listed in Table 2 ▸. The positive Laplacian values typify the closed-shell nature of *peri* Cl⋯Cl interactions.

Three-dimensional deformation and Laplacian maps were plotted to recognize the nature of the intramolecular *peri* interactions. From both deformation maps of individual *peri* interactions it is observed that the lone pairs of Cl atoms signified by the blue electron dense region (supporting information, Fig. S8*a* and S8*c*) are displaced from another. This observation is corroborated in the three-dimensional Laplacian maps which show the VSCCs (valence shell concentrated regions) of one Cl atom is facing the cavity (electron depleted site) of the other Cl atom (supporting information, Figs. S8*b* and S8*d*). This can be interpreted as reduced repulsion between respective atoms which minimizes the strain in the molecule.

#### 3.2.2. NCI analysis   

A NCI (non-covalent interaction) descriptor (Johnson *et al.*, 2010 ▸; Saleh *et al.*, 2012 ▸), based on two scalar field quantities, electron density (ρ) and reduced density gradient (RDG), allows for the extraction of additional information on Cl⋯Cl *peri* interactions. RDG is a dimensionless quantity [*s* = 1/2(3π^2^)^1/3^)|∇ρ|/ρ^4/3^] used in DFT to describe the deviation from a homogeneous electron distribution. Generally non-covalent interactions are characterized by low ρ and low RDG values. The sign of the second largest eigenvalue (λ_2_) of Hessian matrix is utilized to characterize NCI at each RDG isosurface points. Mapping of the sign(λ_2_)ρ(*r*) on RDG isosurfaces categorizes the nature of non-covalent interactions as either stabilizing (λ_2_ < 0) or destabilizing (λ_2_ > 0). Herein the RDG (reduced density gradient) based NCI descriptor is applied to experimental electron density determined from multipole modeling of X-ray data at 100 K.

**Figure 4:**
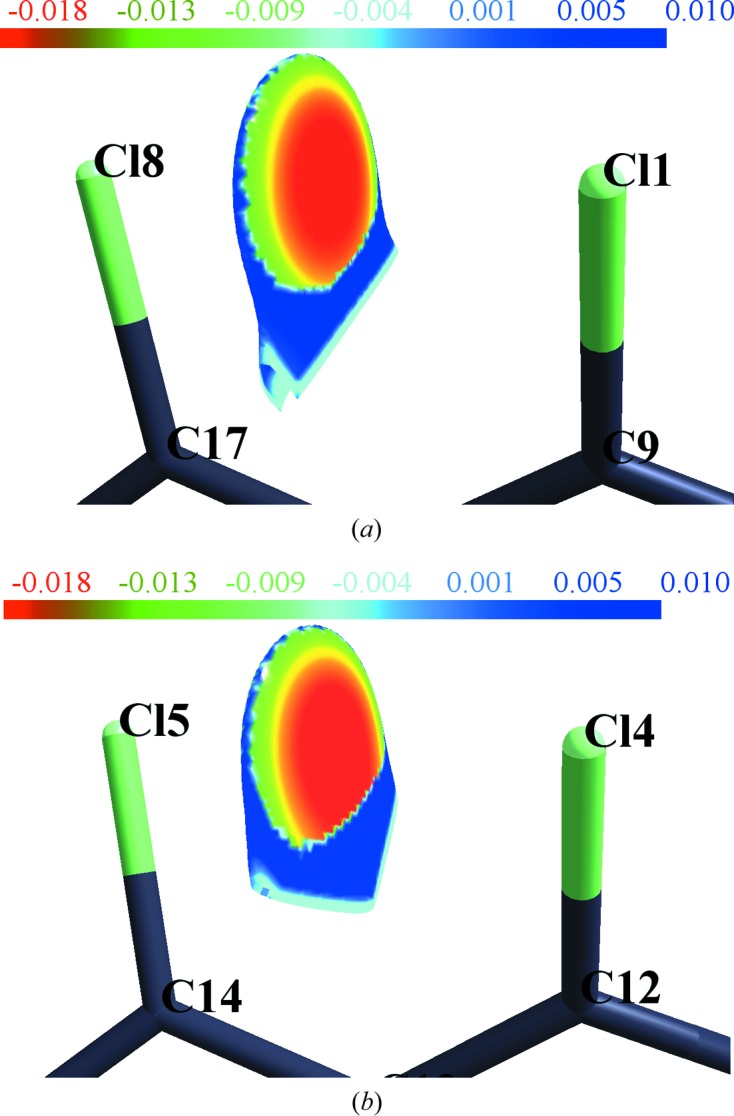
RDG-based NCI isosurfaces with *s* = 0.6 a.u. obtained from the experimental ED model for individual *peri* Cl⋯Cl interactions. The surfaces are coloured on a red–green–blue scale [−0.02 < sign(λ_2_)ρ < 0.015 a.u.]. Red, green and blue indicate stabilizing, intermediate and destabilizing overlap regions, respectively.

The three-dimensional RDG isosurfaces mapped with the sign(λ_2_)ρ(*r*) (Fig. 4 ▸) reflect riveting details about these hitherto unexplored *peri* intramolecular interactions. The three-dimensional surfaces depict the characteristics of all interaction types – attractive (red), dispersive (yellow–green) and repulsive (blue) in the interaction zone between *peri* Cl atoms. The excess of the red region at the centre of the surface implies the dominance of attractive factors over the dispersive (yellow and green area) and repulsive ones (blue region present at the periphery of the surface). Therefore, these observations strengthen the argument provided by the three-dimensional Laplacian maps (supporting information, Figs. S8*b* and *d*). However, the blue region present at the bottom of the surface has contributions from the electron density of the ring critical points (3, +1) (denoted by blue dots in Fig. 3 ▸) where the λ_2_ value is positive. Thus, it can be hypothesized that when the molecule is in an ideal planar conformation, a severe destabilizing interaction between lone pairs of *peri*-substituted Cl atoms dominates. In order to lessen these forces, the molecule adopts a twisted conformation to enable the Cl⋯Cl *peri* interactions to possess excess attractive components over repulsion. It may be claimed that this description from NCI analysis is the first of its kind in the fundamental understanding of steric hindrance between *peri*-substituted groups.

### 3.3. Probe of aromaticity   

The influence on the aromaticity of OCN due to distortions by the overcrowding of Cl atoms is evaluated from NICS (Nuclear Independent Chemical Shift) index and QTAIM theory.

#### 3.3.1. NICS calculation   

The nucleus independent chemical shift (NICS) is a robust and simple technique to examine aromaticity based on the measurement of magnetic shielding due to the ring current produced by π-electron delocalization (Chen *et al.*, 2005 ▸). Negative NICS values (in p.p.m.) refer to a diatropic ring current, suggesting aromaticity, while positive NICS values refer to a paratropic ring current, indicating antiaromaticity. NICS values are calculated by placing a dummy atom at the geometric centre of the ring as well as at 1 Å above the centre. The NICS value at 0 Å [NICS (0)] is essentially dominated by the contribution from the σ bonds in the ring. The value at 1.0 Å above the plane of the molecule [NICS (1 Å)] therefore gives a better measure for examining the aromaticity (Frash *et al.*, 2001 ▸; von Ragué Schleyer *et al.*, 2001 ▸).

In the present study, the calculated values of NICS (1 Å) of each ring marked as *A* and *B* in Table 3 ▸ are compared with the corresponding values calculated for naphthalene at the same level of theory. The NICS (1 Å) value associated with the molecule OCN is less negative compared with that of naphthalene and the consequent percentage difference in NICS (1 Å) values suggests an appreciable loss in aromaticity for the molecule OCN. However, the primary reason for the reduction in aromaticity for OCN could not be deciphered from NICS values as they do not provide information on the preferential accumulation of electron density in the molecular plane due to the formation of a π bond. This can be obtained from ellipticity profile analysis and is discussed as follows.

#### 3.3.2. Ellipticity profiles   

Ellipticity [∊ = (λ_1_/λ_2_)−1] is defined as the ratio of the eigenvalues λ_1_ and λ_2_ of the Hessian matrix that corresponds to the negative curvature of electron density along two perpendicular directions to the bond path as per the QTAIM theory (Bader, 2002 ▸). Evaluation of the ellipticity for the entire bond path rather than at BCPs reveals subtle information of π-bonding effects (Cheeseman *et al.*, 1988 ▸). In the ellipticity profile plots, ϕ_ref_ is defined as the angle between the major axis of the ellipticity, λ_2_ (least negative curvature perpendicular to the λ_3_ along the bond axis) and a reference vector normal to the π-plane. Generally for a homopolar bond near the BCP region, ϕ_ref_ is ideally zero as the two vectors are parallel and at a distance of 0.5 Å approximately from the atomic nuclei, and ϕ_ref_ assumes the value of 90° as the major axis λ_2_ lies in the π-plane.

**Figure 5:**
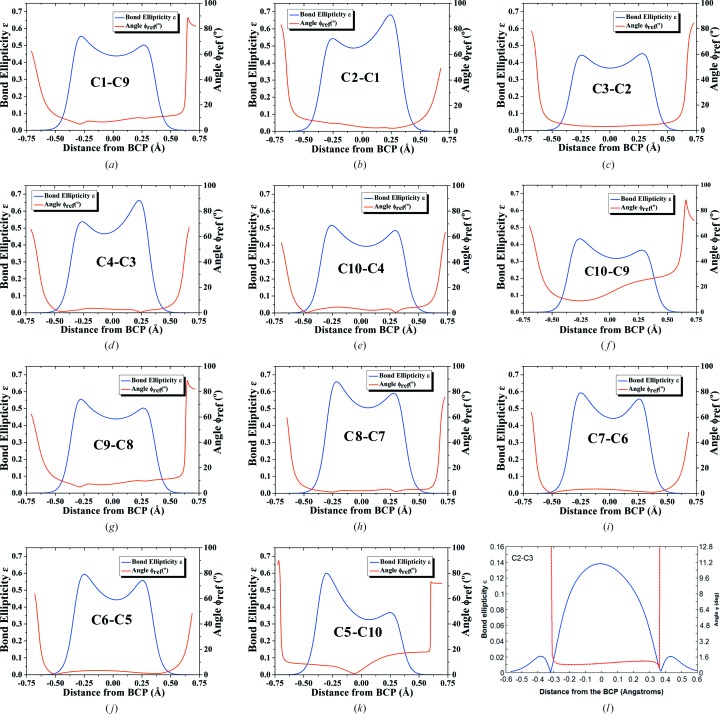
(*a*)–(*k*) Plots of the experimental ellipticity ε along the bond path (solid blue line) with the ϕ_ref_ angle (solid red line) for all 11 C—C covalent bonds in OCN. (l) Ellipticity profile of a naphthalene ring taken from the literature (Farrugia *et al.*, 2009 ▸) indicating aromaticity by showing symmetrical distribution in ellipticity due to delocalization. The reference vector is normal to the plane of the ring in all cases.

Bond ellipticity profiles computed from experimental charge-density analysis of OCN provide an account of nature of π-electron distribution. The common attribute observed in all the 11 C—C bond ellipticity profiles (Fig. 5 ▸ *a*–*k*) is the asymmetric double-hump shape in contrast to a symmetrical one for C—C bonds in naphthalene (Farrugia *et al.*, 2009 ▸; Fig. 5 ▸ *l*). Such behaviour of C—C bonds is somewhat puzzling since for typical C—C aromatic bonds, ∊ is relatively large in the region near the BCPs. A careful investigation of these profiles brings out the peculiarity in the π-electron delocalization between C atoms. The asymmetric double hump signifies the concentration of π-electron density at C centres and the extent of π-electron density concentration varies severely in all the C atoms. This establishes the ‘localized double bond’-like nature in the naphthalene ring instead of a continuous delocalized π-system and can be correlated with the ineffective overlap of *p_z_* orbitals of the respected C atoms. Indeed, this factor is the cause for reduced aromaticity in OCN, also corroborated from NICS values.

### 3.4. Unraveling the differences in dihedral angles   

Having established the nature of *peri* interactions between two pairs of Cl atoms (Cl1⋯Cl8/Cl4⋯Cl5) and its influence on the conformation of the molecule, the unusual difference in dihedral angles (ϕ_1_ = 36.80° and ϕ_2_ = 24.42°, Fig. 2 ▸ *a*) at the *peri* substituted region of OCN needs to be probed.

#### 3.4.1. Geometry optimization   

**Figure 6:**
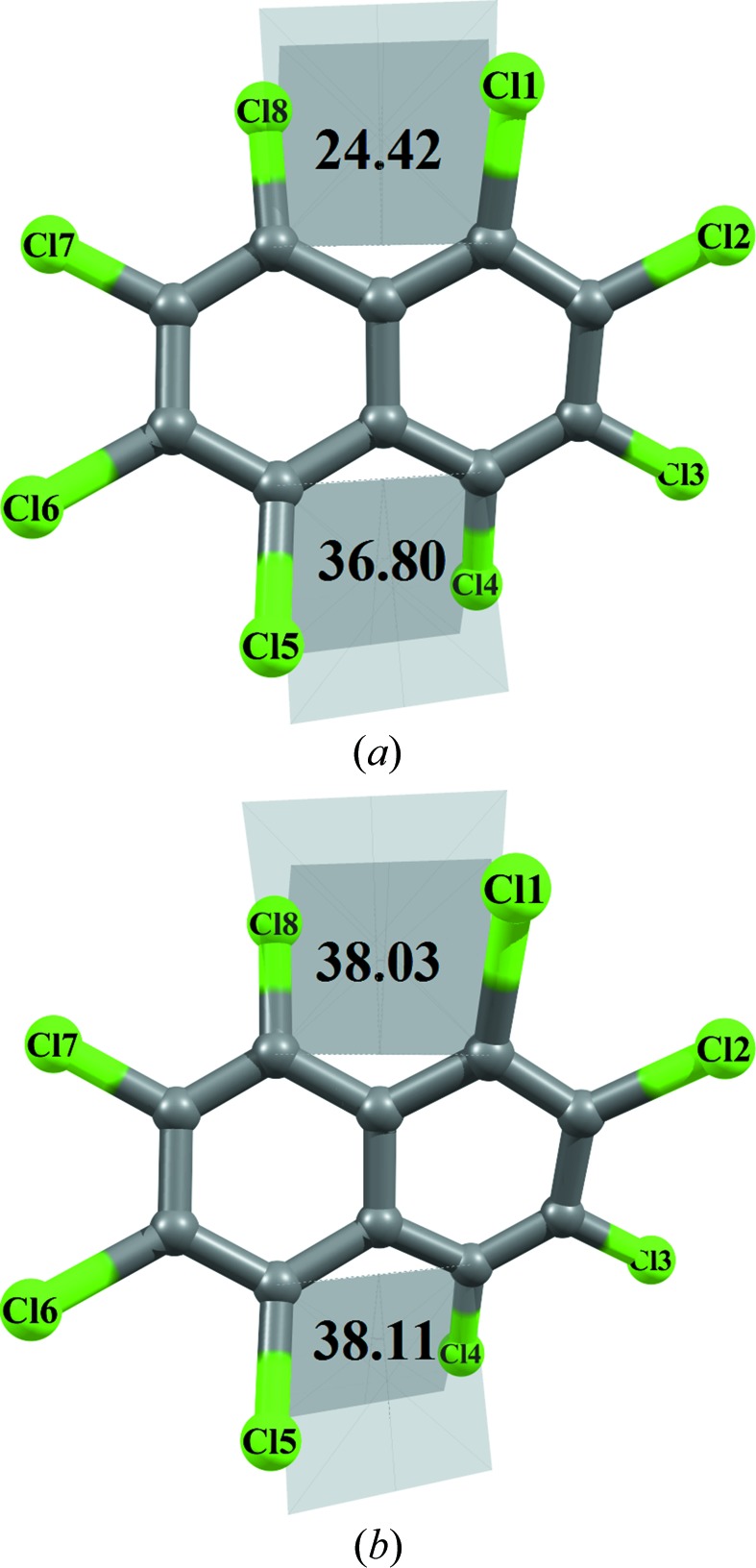
(*a*) Geometry of OCN in the crystalline state. (*b*) Geometry of OCN in the solvated optimized state.

The remarkable difference of 12.38° observed specifically with chlorine substitution in naphthalene has been addressed *via* energy optimization of OCN (taking the initial geometry from crystalline phase minima) using the integral equation formalism (IEF) version of the polarizable continuum solvation model (PCM; Tomasi *et al.*, 2005 ▸) at the wB97XD/6-311+g* level. The result of the outcome is quite noteworthy as there is a significant change in conformation of the overcrowded OCN molecule from the crystal geometry to the optimized geometry in solution (Fig. 6 ▸). The energy difference between the optimized solvated phase and the crystalline phase is −5.26 kJ mol^−1^. The prominent feature observed in the optimized conformer is that the dihedral angles are nearly equal (ϕ_1_ ≃ ϕ_2_ ≃ 38°) at the *peri* substituted positions. This ensures that the dihedral angle ϕ_2_ involving the *peri* interaction between Cl1 and Cl8 reverts from 24.42 to 38.03° in solution.

#### 3.4.2. Electrostatic potential isosurfaces   

**Figure 7:**
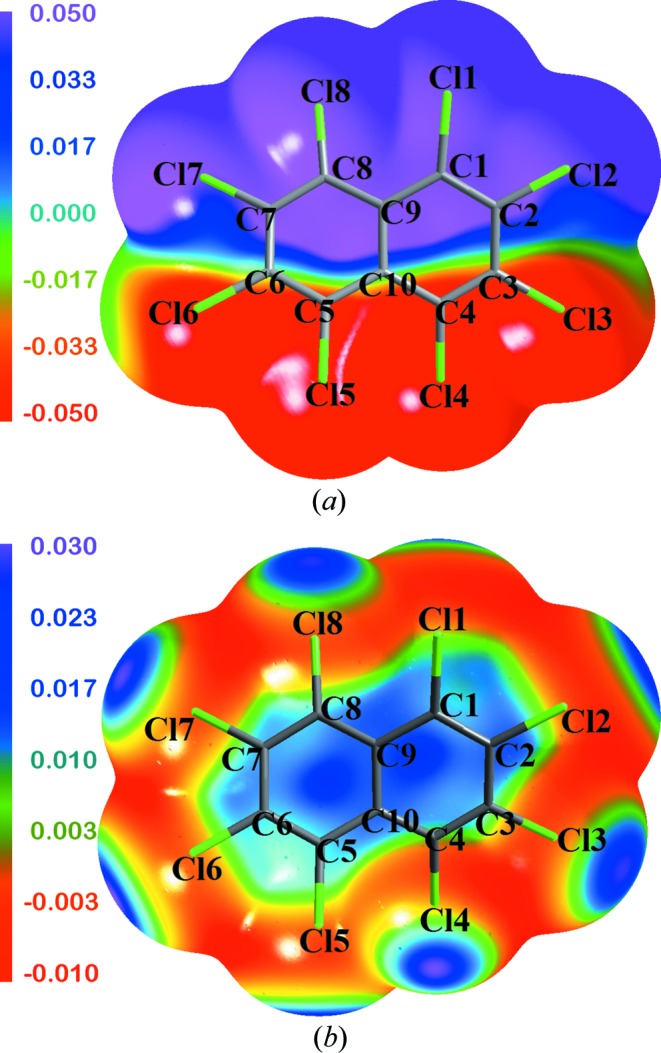
ESP map drawn at the isoelectron density surface at 0.001 a.u. for (*a*) crystalline geometry and (*b*) for solvated optimized geometry.

Molecular electrostatic potentials (MESP) allow for the description of chemical reactivity and assist in exploring molecular aggregation in an isolated phase, whereas in a crystal these surfaces provide perspectives regarding electrostatic complementarity between interacting molecules leading to packing of the molecules in the lattice. The electrostatic potential (ESP) maps are drawn at isodensity surfaces of 0.001 a.u. for both the crystal and solvated optimized geometries of OCN exhibit striking contrast with each other (Fig. 7 ▸). The electropositive isosurface regions are indicated in violet, while the electronegative regions are displayed in red. Due to the symmetric nature of the optimized solvated geometry of the OCN molecule, the electrostatic features on the isosurface are uniform. However, in the crystal geometry the ESP isosurface around the *peri* substituted Cl1 and Cl8 is positive (violet and blue) compared with the negative regions (red) of the other pair of *peri* substituted Cl4 and Cl5. This exceptional observation in the crystal geometry is backed by a similar trend seen in Bader charges (supporting information, Table S6; Bader, 2002 ▸) and the electrostatic potential values calculated at the nuclear sites of each atom from the experimental multipole model (supporting information, Table S7; Volkov *et al.*, 2006 ▸). The inhomogeneity in electrostatic features on the isosurface of the crystal geometry can be mainly attributed to the difference in the dihedral angle at the *peri* substituted region.

#### 3.4.3. Conformational analysis   

An elementary dihedral scan was performed to estimate the energy change in the molecule when individual dihedral angles (ϕ_1_ and ϕ_2_) move from the solvated optimized geometry to the arrested conformation in the crystal lattice using the PCM model in benzene solvent. The energy difference of 2.65 kJ mol^−1^ for ϕ_2_ change from 38.03° to 24.42° at Cl1⋯Cl8 (supporting information, Fig. S9*a*) is much higher than the energy difference of 0.026 kJ mol^−1^ for the ϕ_1_ change from 38.11 to 36.80° at Cl4⋯Cl5 (supporting information, Fig. S9*b*). This analysis establishes the higher destabilizing effect of intramolecular Cl1⋯Cl8 (upper half) *peri* interactions compared with Cl4⋯Cl5 (bottom half). The OCN molecule undergoes a conformational adjustment in the crystalline phase relative to the optimized phase in the benzene solvent. The relatively rigid OCN molecule resorts to in-plane bending of two corresponding pairs of *peri* substituted C—Cl bonds which adjust to the crystal forces, obviously bringing the intermolecular interactions to the fore. Clearly molecular electrostatic potential surfaces activated by the crystal symmetry bring in the differences in intermolecular interactions depending on the top and bottom *peri* Cl⋯Cl contacts.

#### 3.4.4. Influence of intermolecular interactions of individual *peri* Cl atoms on molecular geometry   

Analysis of the role of the individual intermolecular interactions involving the *peri* substituted Cl atoms using a high-resolution charge density analysis is deemed essential to account for the observed difference in dihedral angles. The absence of any type of hydrogen-bonding motifs in OCN makes this study quite fascinating. The topological analysis of ρ(*r*) illustrates (3,−1) BCPs for Cl⋯Cl and Cl⋯π as two major intermolecular interactions present in the crystal structure.

**Figure 8:**
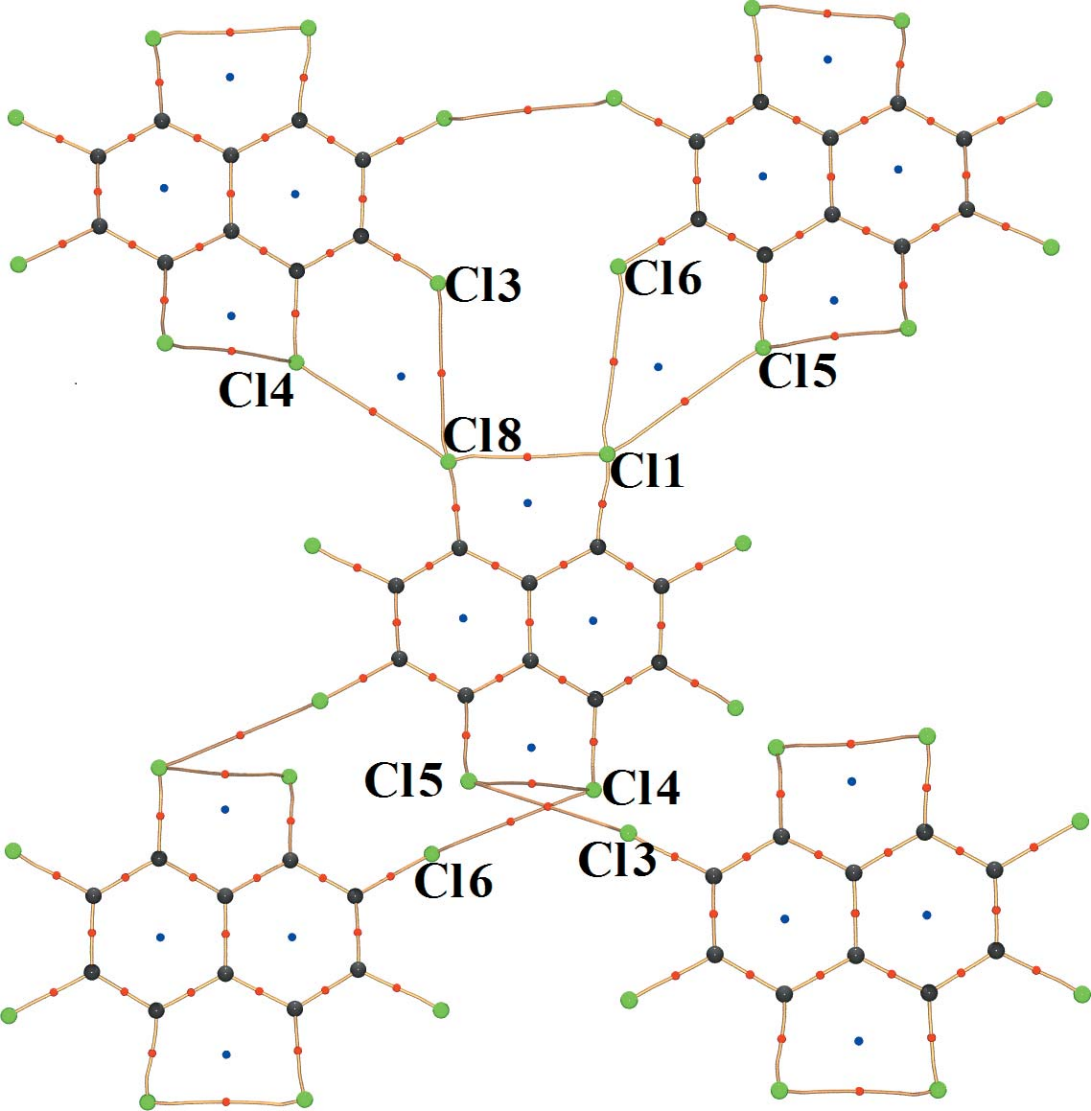
Molecular graph depicting the various intermolecular Cl⋯Cl interactions involving (*a*) *peri* interaction pair Cl1 and Cl8; (*b*) *peri* interaction pair Cl4 and Cl5.

**Figure 9:**
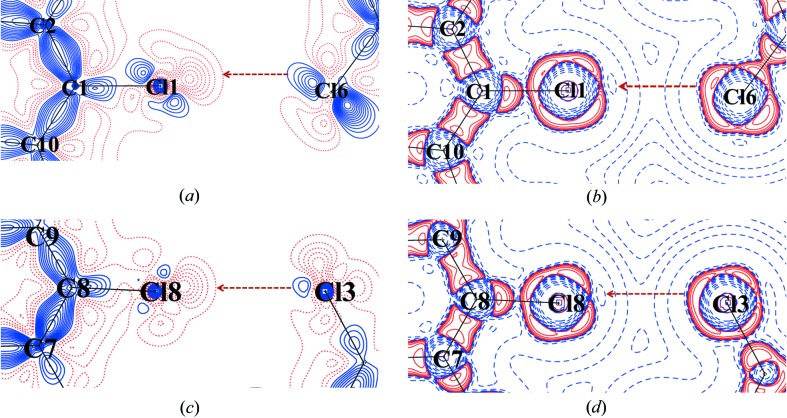
(*a*) Two-dimensional deformation density; (*b*) two-dimensional Laplacian plot of the intermolecular Cl1⋯Cl6 interaction region; (*c*) two-dimensional deformation density; (*d*) two-dimensional Laplacian plot of the intermolecular Cl8⋯Cl3 interaction region. Blue (solid lines) and red (broken lines) colours represent positive and negative contours, respectively (reversed in case of Laplacian). Contours are drawn at intervals of ± 0.05 e Å^−3^. The Laplacian is plotted on logarithmic contours.

From the molecular graph (Fig. 8 ▸) it is quite evident that the nature of Cl⋯Cl interactions associated with the *peri* interacting pair Cl1/Cl8 is strikingly different from that of Cl4/Cl5 (shown as red circles). Cl1 and Cl8 participate in typical type II halogen bonding (Bui *et al.*, 2009 ▸; Hathwar & Row, 2010 ▸) with Cl6 and Cl3, respectively [Cl1⋯Cl6 = 3.460 (2) Å, ∠C1—Cl1⋯Cl6 = 176.08°; Cl3⋯Cl8 = 3.514 (3) Å, ∠C9—Cl8⋯Cl3 = 174.54°]. The near linearity in the angles indicates conventional electrophile-nucleophile pairing. However, although the linearity is retained, the distances hover around the van der Waals limit (3.6 Å) preventing a robust overlap of electron density due to the interference of Cl4 and Cl5, which enter the interaction sphere. Figs. 9 ▸ and 10 ▸ show the two-dimensional deformation and Laplacian maps depicting the intermolecular space associated with the *peri* pairs Cl1/Cl8 and Cl4/Cl5, respectively. The σ-hole (red dots) along the C—Cl bond axis facing the lone pair density (blue solid) on the Cl atom is depicted in Figs. 9 ▸(*a*) and (*c*). The two-dimensional Laplacian plots (Fig. 9 ▸ *b* and *d*) characterize the ‘polar flattening’ on the respective Cl atoms in accordance with the lump (red solid)–hole (blue dash) interaction at the valence shell charge concentration (VSCC) region (Politzer *et al.*, 2013 ▸; Politzer & Murray, 2013 ▸).

**Figure 10:**
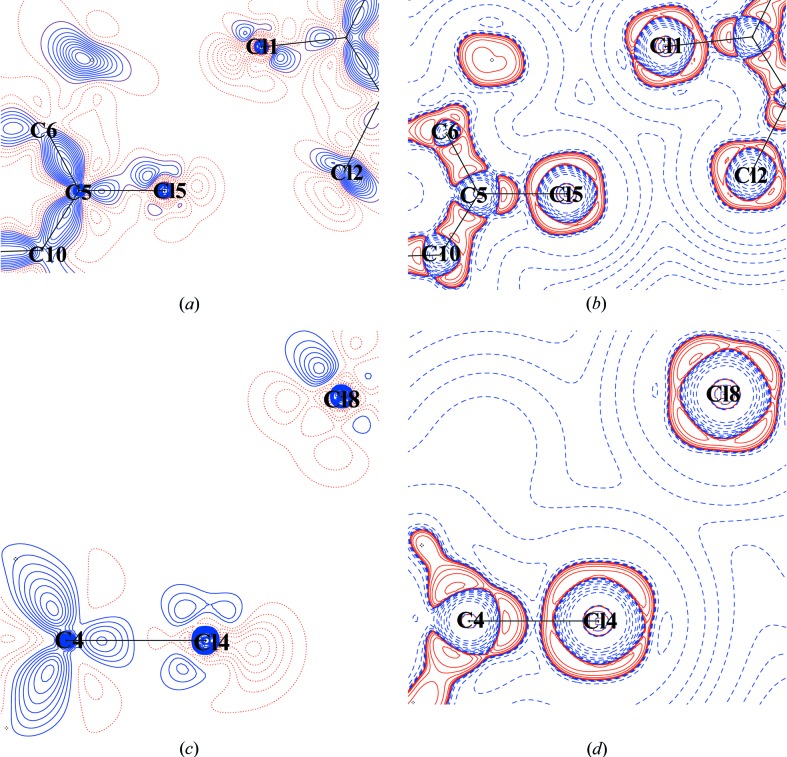
(*a*) Two-dimensional deformation density; (*b*) two-dimensional Laplacian plot of the intermolecular Cl5⋯Cl4 interaction region; (*c*) two-dimensional deformation density; (*d*) two-dimensional Laplacian plot of the intermolecular Cl4⋯Cl8 interaction region. Blue (solid lines) and red (broken lines) colours represent positive, negative contours respectively (reversed in case of Laplacian). Contours are drawn at intervals of ± 0.05 e Å^−3^. The Laplacian is plotted on logarithmic contours.

On the other hand, the other *peri* pair, Cl4 and Cl5 interacts with Cl8 and Cl1 in a *trans* type I Cl⋯Cl contact as evident from the interaction geometry [Cl1⋯Cl5 = 3.4780 (3) Å, ∠C1—Cl1⋯Cl5 = 129.99°, ∠C6—Cl5⋯Cl1 = 123.90°, Cl8⋯Cl4 = 3.4615 (2) Å, ∠C9—Cl8⋯Cl4 = 131.63°, ∠C4—Cl4⋯Cl8 = 119.18°]. The corresponding two-dimensional deformation and Laplacian maps (Figs. 10 ▸ *a*–*d*) show that the charge-depleted (CD) regions of Cl atoms are directed towards each other, a phenomenon indicative of reduced repulsion.

In addition, atom Cl4 interacts with Cl6 in a quasi type I/type II contact (Mukherjee *et al.*, 2014 ▸) [Cl4⋯Cl6 = 3.5316 (3) Å, ∠C4—Cl4⋯Cl6 = 115.83°, ∠C7—Cl6⋯Cl4 = 136.99°; supporting information, Fig. S10] which is weak. Furthermore, atom Cl5 with its nucleophilic character participates in an ineffective type II contact with Cl3 [Cl5⋯Cl3 = 3.5316 (3), ∠C6—Cl5⋯Cl3 = 118.78°, ∠C3—Cl3⋯Cl5 = 162.51°; supporting information, Fig. S11].

**Figure 11:**
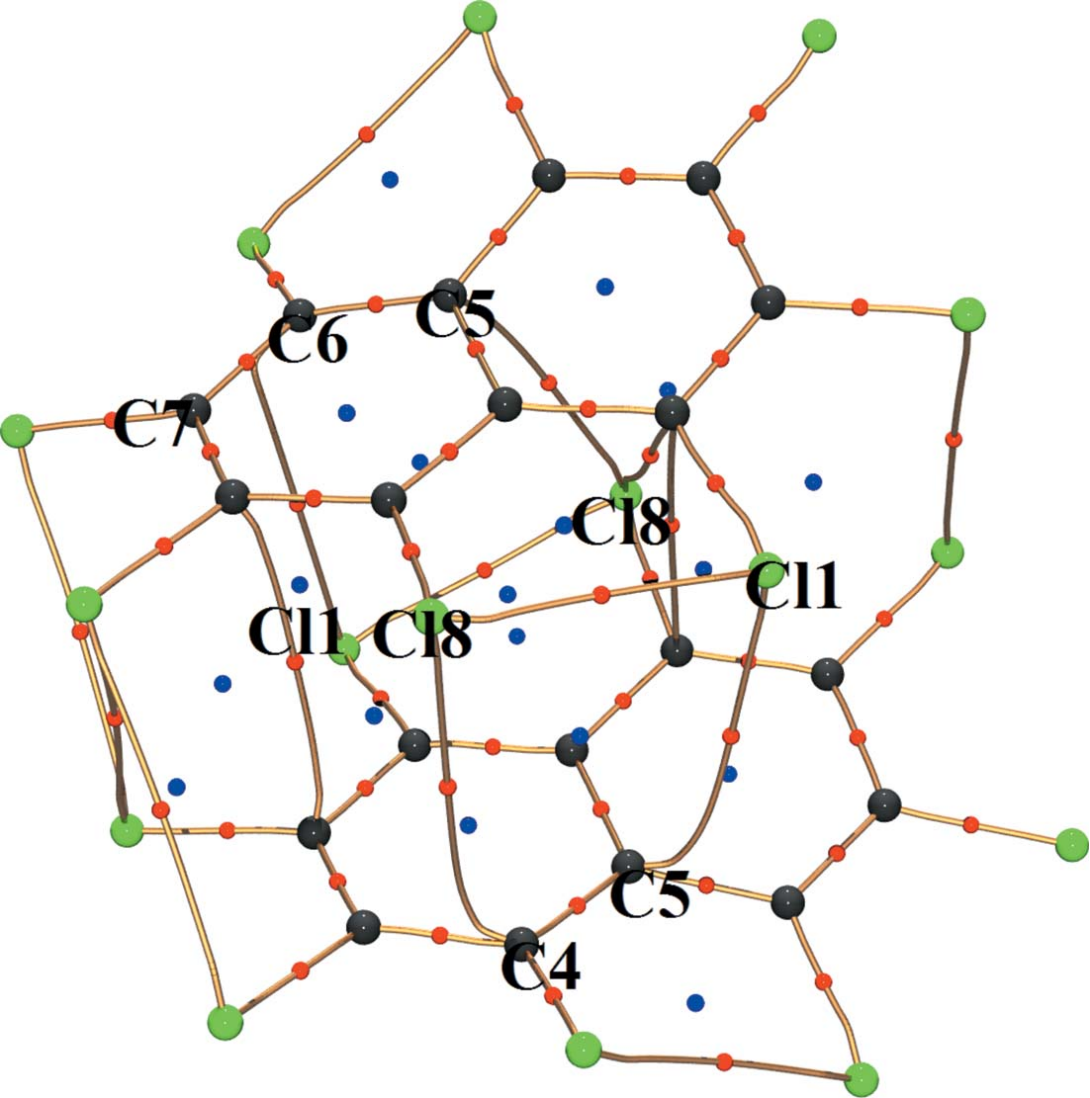
Molecular graph showing various intermolecular interactions between molecular units packed along the 2_1_ screw axis parallel to the *b* axis. Only the *peri* interacting pair Cl1 and Cl8 exhibits Cl⋯π interactions.

It is notable that Cl⋯π contacts are exclusively found to be associated with the *peri*-pair Cl1/Cl8 as can be seen from the molecular graph (Fig. 11 ▸). These occur between molecular units packed along the 2_1_ screw axis parallel to the *b* axis. It can be surmised that the differences in top and bottom dihedral angles in the OCN molecule might be a consequence of such Cl⋯π contacts limited to one half of the molecule.

#### 3.4.5. Interaction energies   

**Figure 12:**
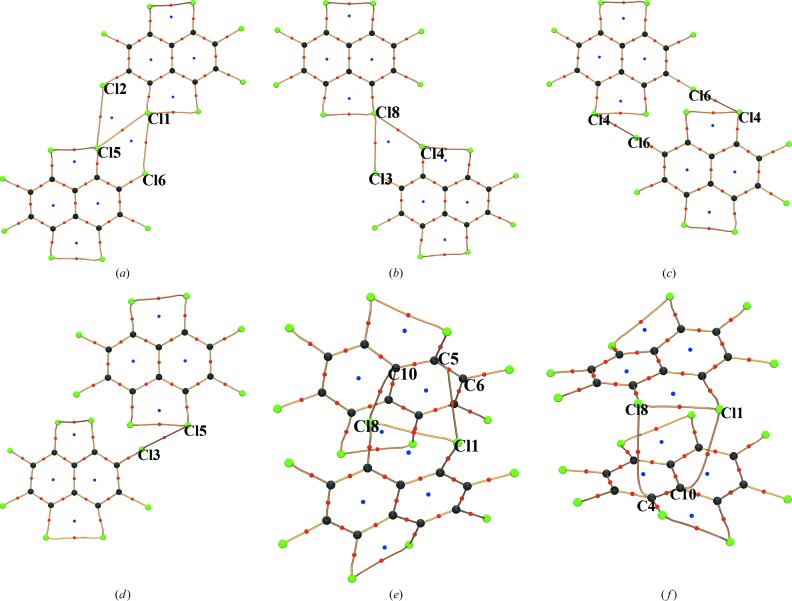
Molecular graphs showing intramolecular and various intermolecular bond paths of different dimers in the crystal structure.

In order to evaluate the contribution of the intermolecular Cl⋯Cl and Cl⋯π interactions involving the *peri* substituted Cl atoms towards crystal packing, the crystal structure of OCN is dissected into six dimers (Fig. 12 ▸). The electrostatic contribution to the interaction energy of the individual dimers is calculated from the exact potential/multipole moment hybrid method (EPMM; Volkov *et al.*, 2004 ▸). The values are listed in Table 4 ▸ corresponding to individual dimers. The positive electrostatic energies obtained for dimers (III) and (IV) support the earlier observation that the interactions involving atoms Cl4⋯Cl6 and Cl3⋯Cl5 are weak and can be considered only as structure-directed contacts. Indeed, the negligible energy rise in the molecule due to the change in ϕ_1_ (supporting information, Fig. S9*a*) from the optimized solvated geometry to the crystalline phase is in support of this observation. On the other hand, the electrostatic energies associated with dimer (I) and dimer (II), pertaining to the *peri* substituted pair Cl1 and Cl8, are substantially negative, thus validating the attractive type (II) intermolecular Cl⋯Cl interactions.

Pairwise interactions between molecules in dimers (V) and (VI) organized by several interactions including Cl⋯π results in a highly negative electrostatic interaction energy. The resulting supramolecular assembly thus compensates for the destabilizing *peri* interactions between Cl1 and Cl8. This concurs with the somewhat steep energy rise in the molecule (2.65 kJ mol^−1^) due to the change in ϕ_2_ (supporting information, Fig. S9*b*) from the optimized solvated geometry to the crystalline phase. It is to be noted that the electrostatic energy associated with the Cl⋯π interaction alone is not discernible from this analysis. Hence it is still uncertain from these interaction energy values whether the Cl⋯Cl or Cl⋯π interaction is causing the in-plane bending of C—Cl bonds that leads to asymmetry in the crystal geometry of OCN.

#### 3.4.6. Trimer optimization   

**Figure 13:**
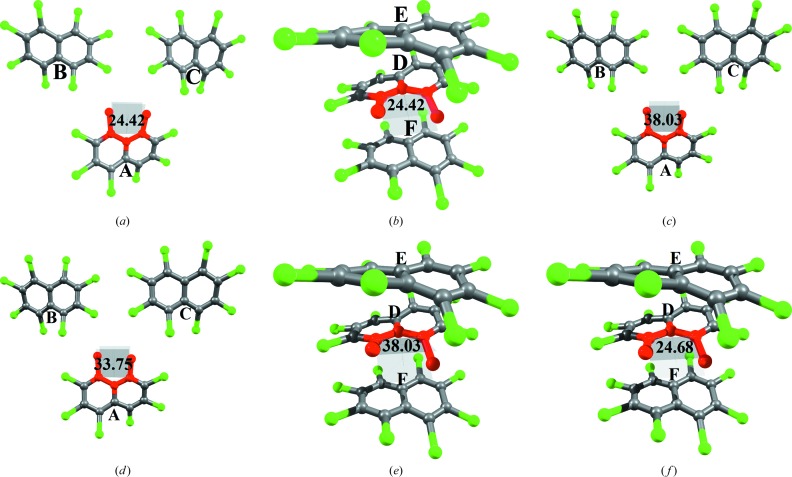
(*a*)–(*b*) Molecular conformations from crystal geometry *T*1° and *T*2°. (*c*)–(*d*) Molecular conformations showing the value of ϕ_2_ in a starting geometry (*T*1) and optimized geometry (*T*1′). (*e*)–(*f*) Molecular conformations showing the value of ϕ_2_ in the starting geometry (*T*2) and optimized geometry (*T*2′).

To settle this issue, restricted gas-phase optimization of a pair of trimers, *T*1 and *T*2 (Fig. 13 ▸), was performed at the wB97XD/6-31g(d) level. Restricted optimization was carried out to mimic the environment in a condensed phase. T1 involves the *peri* substituted pair Cl1/Cl8 in molecule *A* interacting with molecules *B* and *C* primarily through intermolecular Cl⋯Cl interactions, whereas in *T*2, molecule *D* is sandwiched between *E* and *F* forming Cl⋯π interactions.

Initial coordinates of all atoms for both *T*1 and *T*2 are from the multipole refined structural values (*T*1° and *T*2°, Figs. 13 ▸ *a*–*b*), except that the dihedral angle ϕ_2_ associated with molecules *A* and *D* are fixed corresponding to the optimized solvated geometry value (38.03°). Thus, during optimization which includes only those atoms (Cl1, C1, C10, C9, Cl8) marked in red are allowed to change in both *T*1 and *T*2 (Fig. 13 ▸ *c* and 13*e*). This approach is expected to allow for the study of the respective control through Cl⋯Cl and Cl⋯π interactions in directing the molecules *A* and *D*, respectively, close to planarity by causing in-plane bending of exocylic C—Cl bonds. The optimized conformations of *T*1 and *T*2 are referred to as *T*1′ and *T*2′, respectively (Fig. 13 ▸ *d* and 13*f*). Upon optimization of *T*1 (Fig. 13 ▸ *a*), ϕ_2_ reduces marginally to 33.75° in *T*1′ (Fig. 13 ▸ *b*), whereas in *T*2 optimization, ϕ_2_ results in a value of 24.68° in *T*2′ (Fig. 13 ▸ *d*). It is remarkable to observe that the value matches the final crystal geometry value ϕ_2_ (24.42°, Fig. 2 ▸ *a*), confirming the exclusive role of Cl⋯π in the control of crystal geometry.

This observation can be justified by the following arguments. The angular geometries of the individual type II interactions in the following trimers – *T*1°, *T*1, *T*1′ are listed in Table 5 ▸. The reduced directionality (deviation from linearity, θ_1_ = 166.53°, θ_2_ = 165.30°) associated with a starting geometry of *A* in *T*1 hampers effective type II Cl⋯Cl interactions with *B* and *C*. (The angular geometries of the type II Cl⋯Cl interactions in trimer *T*1 is shown in the supporting information, Fig. S12, the corresponding angular geometries of the type II Cl⋯Cl interactions in the crystal geometry of trimer *T*1° and optimized geometry of trimer *T*1′ are shown in the supporting information, Figs. S13 and S14, respectively.) As a result, the σ-hole interaction involving the nucleophilic Cl atoms in *B* and *C* is unable to drive the in-plane bending of the corresponding Cl1 and Cl8 close to the crystal geometry. On the contrary, the Cl⋯π interactions, being non-directional in nature, are more effective in causing the in-plane deviations of the exocyclic C—Cl bonds and hence they control the crystal packing. Additionally, under these changes, the type II Cl⋯Cl interactions benefit as they are ushered closer to the crystal geometry as seen in *T*2′. This rationalizes the symbiotic relation between Cl⋯π and type II Cl⋯Cl interactions, which induces crystal packing by perturbing the molecular geometry of OCN and consequently the asymmetry becomes incorporated in the molecular shape.

## 4. Conclusions   

The present work documents a unique quantitative estimate of the influence of intra- and intermolecular interactions in an overcrowded molecule, OCN, in controlling the molecular conformation. A hitherto less explored intramolecular interaction between *peri*-substituted halogen atoms which contributes to the steric hindrances is characterized from Bader topological analysis. The RDG-based NCI descriptor applied to multipole electron densities reveals interesting details about *peri* interactions; the confronting lone-pair density reorganizes in such a way that the interaction zone has more attractive than repulsive features so that the strain in the molecule is minimized, a description so far missing in the literature. Perhaps the formation of an α-substituted product in nucleophilic substitution reactions as opposed to the β-product in OCN can thus be attributed to the reduction in steric forces, contributed primarily by the *peri* interactions, in transition state geometry. Additionally we have described the impact of these steric hindrances in imparting localized character to the π electron density. The reduced aromatic character in the naphthalene ring of OCN due to this phenomenon of localized π-electrons is estimated from NICS calculation.

Understanding of the discrepancy in dihedral angles in the crystal phase pinpoints the individual role of major intermolecular interactions (Cl⋯π and Cl⋯Cl) against the opposing *peri* interactions in stabilizing the high-energy conformer. This study brings out the novelty in gauging the modification in electron density of overcrowded molecules when they undergo a transition from the solvated to the crystalline phase and hence the findings from this study could be further extended in establishing the relationship between solid-state properties of organic functional materials (for example, semiconductors, fullerenes) and crystal structures.

## Supplementary Material

Crystal structure: contains datablock(s) ocn. DOI: 10.1107/S205225251601808X/lc5069sup1.cif

Structure factors: contains datablock(s) ocn. DOI: 10.1107/S205225251601808X/lc5069ocnsup2.fcf

Quality of multipole modeling, Computational details, multipole population table, Molecular packing diagram of Octachloronaphthalene (OCN) viewed with the direction of dipole moment vector, Electrostatic Potential calculated at the nuclear sites of each atom, Topological features obtained for all covalent bonds. DOI: 10.1107/S205225251601808X/lc5069sup3.pdf

## Figures and Tables

**Table 1:** Crystallographic parameters

CCDC No.	1479507	(sin θ/λ)_max_ (Å^−1^)	1.098
Molecular formula	C_10_Cl_8_	Reflns collected	105 141
Formula weight	403.70	Unique reflns	13 899
Crystal system	Monoclinic	Completeness (%)	99.5
Space group	*P*2_1_/*n*	Redundancy	8
*a* (Å)	9.7188 (1)	*R*_int_	0.049
*b* (Å)	7.1598 (1)	Spherical atom refinement	
*c* (Å)	18.2787 (3)	*R*_1_ (*F*)	0.046
α (°)	90	*wR*_2_ (*F* ^2^)	0.078
		*w*_1_, *w*_2_	0.0266, 0.1112
β (°)	98.358 (1)	Goodness-of-fit	1.05
γ (°)	90	Δρ_min_, Δρ_max_ (e Å^−3^)	−0.62, 0.76
*V* (Å^3^)	1258.41 (3)	Multipole refinement	
*Z*	4	Reflns used [*I* > 2σ(*I*)]	10581
ρ_calc_ (g cm^−3^)	2.131	No of parameters	563
*F*(000)	784	*R*_1_ (*F* ^2^)	0.024
μ (mm^−1^)	1.758	*wR*_2_ (*F* ^2^)	0.048
		*w*_1_, *w*_2_	0.0262, 0.0481
*T* (K)	100 (2)	Goodness-of-fit	1.003
λ (Å)	0.71073	Δρ_min_, Δρ_max_ (e Å^−3^) all data/sin θ/λ ≤ 0.8 Å^−1^	−0.44, 0.47/ −0.27,0.24

**Table 2:** Topological values of *peri* Cl⋯Cl interactions at (3,−1) BCPs

Interaction	*R_ij_* (Å)	ρ (e Å^−3^)	∇^2^ρ (e Å^−5^)	∊	*G* (kJ mol^−1^ bohr^−3^)	*V* (kJ mol^−1^ bohr^−3^)	|*V*|/|*G*|
Cl1⋯Cl8	2.9941	0.12	1.51	0.13	36.54	−31.95	0.87
Cl4⋯Cl5	3.0255	0.13	1.56	0.24	38.75	−35.01	0.93

**Table 3:** Dissected NICS (in p.p.m.) at ring centres and 1 Å above

Compound	NICS(0)	NICS(1 Å)	NICS(0)	NICS(1 Å)
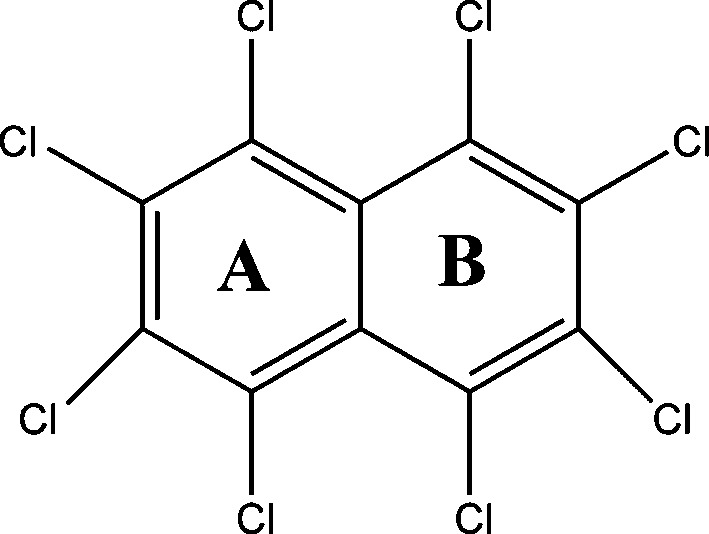	*A*		*B*	
	−8.7	−8.8	−8.4	−8.3
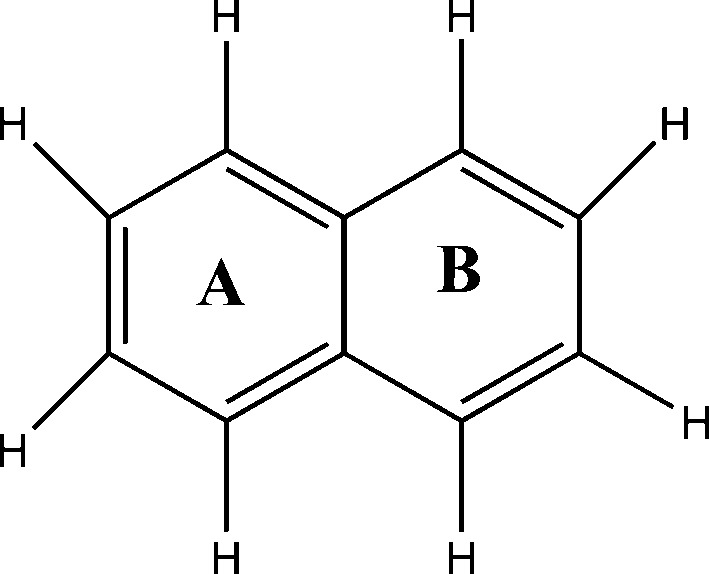	−7.9	−10.8	−7.9	−10.9
% difference in NICS (1 Å) values	18.5		23.9	

**Table 4:** Electrostatic energies of various Cl⋯Cl intermolecular interactions of selected molecular dimers (kJ mol^−1^) in OCN

Dimer	(I)	(II)	(III)	(IV)	(V) and (VI)
Energy (kJ mol^−1^)	−26.4	−31.2	11.6	7.03	−60.6

**Table 5:** Angular geometry (°) of individual type II Cl⋯Cl interactions in all the trimers

Trimer	θ_1_ = ∠C1—Cl1⋯Cl6	θ_2_ = ∠C9—Cl8⋯Cl3
*T*1°	176.08	174.54
*T*1	166.53	165.30
*T*1′	169.09	167.85
