# Supplementary material for: A heuristic approach to evaluate peri interactions versus intermolecular interactions in an overcrowded naphthalene
Source: IUCrJ. 2017 Jan 1;4(Pt 1):37–49. doi: 10.1107/S205225251601808X (PMC5331464; doi:10.1107/S205225251601808X)
Supplement: Supplementary file 3 [file m-04-00037-sup3.pdf]

IUCrJ

Volume 4 (2017)

Supporting information for article:

A heuristic approach to evaluate *peri* interactions vs. intermolecular interactions in an overcrowded naphthalene

Sounak Sarkar and Tayur N. Guru Row

A heuristic approach to evaluate *peri* interactions vs. intermolecular interactions in overcrowded naphthalene

Sounak Sarkar[a] and Tayur Narasingarao Guru Row[a]*

[a]Solid State and Structural Chemistry Unit, Indian Institute of Science, C.V. Raman Avenue, Bangalore, Karnataka, 560012, India

Correspondence email: ssctng@sscu.iisc.ernet.in

Supporting information

S1.: Quality of multipole modeling

S2.: Computational details

S2.1.: Periodic DFT calculation

S2.2.: NCI Analysis

S2.3.: NICS

S2.4.: Geometry optimization

S2.5.: Energy vs. dihedral angle scans

Figure S1: (a) Scatter plot depicting the variation of F_{obs} with F_{cal}. (b) Variation of F_{obs}^2/F_{cal}^2 with (sinθ)/λ.

Figure S2: 3D residual density plot at ±0.2 eÅ^{-3} contour intervals

Figure S3: Fractal dimension plot for data (sinθ/λ≤0.8Å^{-1})

Figure S4: Cl•••Cl Interaction motifs of top half of OCN

Figure S5: Cl•••Cl Interaction motifs of bottom half of OCN

Figure S6: Histogram showing a total of 421 hits having intramolecular Cl•••Cl interactions

Figure S7: Histogram of *peri* Cl•••Cl distance showing maximum case around 3±0.025Å

Figure S8: 3D deformation density; (b) 3D Laplacian plot of the Cl(1)•••Cl(8) interaction region; (c) 3D deformation density and (d) 3D Laplacian plot of the Cl(4)•••Cl(5) interaction region. Blue represents charge concentration (CC) and red represents

IMPORTANT: this document contains embedded data - to preserve data integrity, please ensure where possible that the IUCr Word tools (available from http://journals.iucr.org/services/docxtemplate/) are installed when editing this document.

charge depletion (CD) in deformation maps drawn at the intervals of ±0.08 e$Å^{-3}$. 3D Laplacian isosurfaces is plotted at -17.5e$Å^{-5}$.

Figure S9: (a) Potential energy scan for dihedral angle ϕ_2 (b) Potential energy scan for dihedral angle ϕ_1

Figure S10: (a) 2D deformation density; (b) 2D Laplacian plot of the intermolecular Cl(4)•••Cl(6) interaction region. Blue (solid lines) and red (broken lines) colors represent positive, negative contours respectively (reversed in case of Laplacian).Contours are drawn at the intervals of ±0.05 e $Å^{-3}$. Laplacian is plotted on logarithmic contours.

Figure S11: (a) 2D deformation density; (b) 2D Laplacian plot of the intermolecular Cl(5)•••Cl(3) interaction region. Blue (solid lines) and red (broken lines) colors represent positive, negative contours respectively (reversed in case of Laplacian).Contours are drawn at the intervals of ±0.05 e $Å^{-3}$. Laplacian is plotted on logarithmic contours.

Figure S12: Molecular conformations showing the angular geometries of the type II Cl•••Cl interactions in the geometry of trimer T1

Figure S13: Molecular conformations showing the angular geometries of the type II Cl•••Cl interactions in the optimized geometry of trimer T1′.

Figure S14: Molecular conformations showing the angular geometries of the type II Cl•••Cl interactions in the optimized geometry of trimer T1°.

Figure S15: (a) Plot of RDG (reduced density gradient) vs. electron density (ρ). (b) Plot of RDG (reduced density gradient) vs. electron density electron density multiplied by the sign of the second Hessian eigen value (ρ*sign(λ_2)) for *peri* interaction between Cl(1)•••Cl(8).

Figure S16: (a) Plot of RDG (reduced density gradient) vs. electron density (ρ). (b) Plot of RDG (reduced density gradient) vs. electron density electron density multiplied by the sign of the second Hessian eigen value (ρ*sign(λ2)) for *peri* interaction between Cl(4)•••Cl(5).

Figure S17: Molecular packing diagram of Octachloronaphthalene (OCN) viewed along b axis. The right arrow indicates the direction of dipole moment vector. Blue indicates the c axis, red indicates the a axis, and green indicates the b axis.

Figure S18: (a) Comparison of ρ values for all covalent bonds obtained from experiment and theory. (b) Comparison of $\nabla^2\rho$ values for all covalent bonds obtained from experiment and theory.

Table S1: Monopole Populations, Radial Parameters and Net Atomic Charges

Table S2: Dipole Population Parameters.

Table S3: Quadrupole Population Parameters

Table S4: Octupole Population Parameters.

Table S5: Hexadecapole Population Parameters

Table S6: Aim charge of the individual atoms derived from the experimental multipole modeled electron density.

Table S7: Electrostatic Potential calculated at the nuclear sites of each atom.

Table S8: Topological features obtained for all covalent bonds in OCN. r_1 and r_2 are the distances from the BCP to the first atom (A) and second atom (B), respectively. The interaction length, $R_{ij} = (r_1 + r_2)$. The values obtained from periodic calculations using the M062X/TZVP method are given in italics.

S1. Quality of multipole modeling

The topological features of the intra and intermolecular interactions have been explored on the basis of multipole modeling on experimental structure factors using the Hansen−Coppens formalism. The Hirshfeld rigid bond (Hirshfeld, 1976) test applied to all covalent bonds validates the quality of the multipole model after the final cycle of refinement. The C(9)-Cl(8) single bond is found to have the largest difference of mean square displacement amplitude (DMSDA) value of 4×10^{-4} Å^2. The residual electron density peaks are -0.44 and 0.47e Å^{-3} with an RMS value of 0.09 eÅ^{-3} for minimum and maximum values, respectively at full resolution (1.08 Å^{-1}, Fig. S4). The fractal dimension plot (Meindl & Henn, 2008) (Fig S5) which provides the overall distribution of residual electron density in the unit cell is symmetric in nature and parabolic in shape ($\sin\theta/\lambda \leq 0.8$Å^{-1}).

S2. Computational details

S2.1. Periodic DFT calculation

Positional parameters obtained from the experimental charge density model have been used for density functional calculations using the hybrid exchange correlational functional M062X (Zhao & Truhlar, 2008) with TZVP (Schäfer *et al.*, 1992; Peintinger *et al.*, 2013) basis set included in CRYSTAL14 package (Dovesi *et al.*, 2013).The shrinking factors (IS1, IS2, and IS3) and the reciprocal lattice vectors were set to 4 (with 30 k-points in irreducible Brillouin zone). The

bielectronic Coulomb and exchange series values for the truncation parameter were set as ITOL1-ITOL4 = 7 and ITOL5 = 14, respectively, for the calculations. The level shifter was set to 0.3 Hartree/cycle as 30% mixing of Fock/KS matrices (FMIXING) given in the input. An SCF convergence limit of the order of 10^{-6} Hartree was used. In the static model, atomic thermal displacement parameters for all atoms were set to zero. Structure factors were calculated for a resolution of 1.11$Å^{-1}$, which were used for the theoretical multipolar model. Refinements and analysis for the theoretical charge density model were performed using the XD software package following the same methodology used for the experimental charge density modeling.

S2.2. NCI Analysis

NCImilano (Saleh *et al.*, 2013) has been performed with the rho (electron density) and gradrho (gradient of electron density) grid files obtained from experimentally modeled electron density. It generated RDG and ρ*sign (λ_2) cube files with rho (r) cutoff value 0 to 0.05 au. Reduced density gradient (RDG) isosurface value was generated at 0.6 au and plotted in MoleCoolQt software (Hübschle & Dittrich, 2011). The color scale of RDG surfaces is -0.02< r < 0.015 au.

S2.3. NICS

The nucleus independent chemical shift (NICS) has been calculated using the GAUSSIAN09 (Frisch *et al.*, 2009) package by the GIAO (London, 1937; Cheeseman *et al.*, 1996) method at the M062X/6-311+g* level of density functional theory.

S2.4. Geometry optimization

Energy optimization (taking the initial geometry from crystalline phase minima) was performed using the integral equation formalism (IEF) version of the polarizable continuum solvation model (PCM) (Tomasi *et al.*, 2005) at wB97XD (Chai & Head-Gordon, 2008)/6-311+g* level to examine the dielectric field effect of crystallizing solvent (benzene) on the optimized conformation of OCN.

S2.5. Energy vs. dihedral angle scans

The ϕ_1(Cl(4)-C(4)-C(6)-Cl(5) dihedral angle was varied, and energy was estimated at the interval of -0.0524° in 25 steps, allowing the geometry to be relaxed at each point of f_1. In case of ϕ_2(Cl(1)-C(1)-C(9)-Cl(8), energy was estimated at the interval of -0.5444° in 25 steps. Calculations were performed using PCM at wB97XD/6-311+g* level.

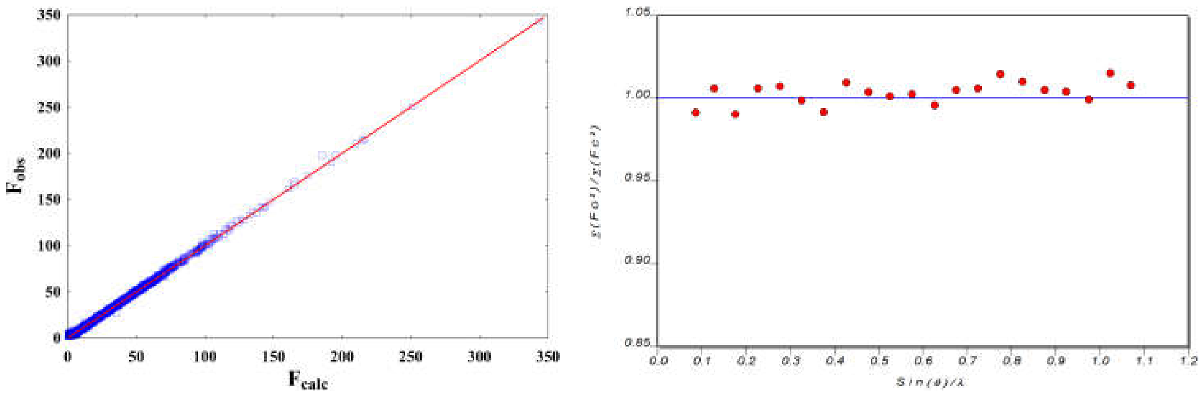

(a) (b)

Figure S1 (a) Scatter plot depicting the variation of F_{obs} with F_{cal}. (b) Variation of F_{obs}^2/F_{cal}^2 with (sinθ)/λ.

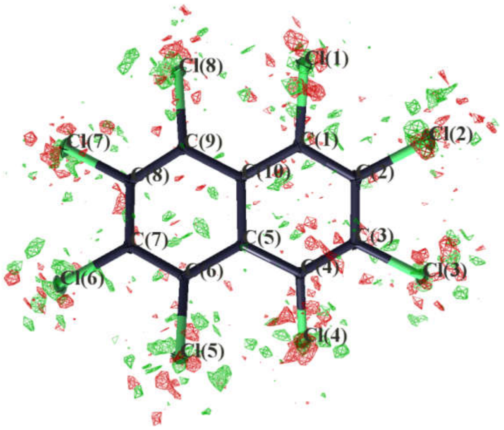

Figure S2 3D residual density plot at ±0.2 $eÅ^{-3}$ contour intervals

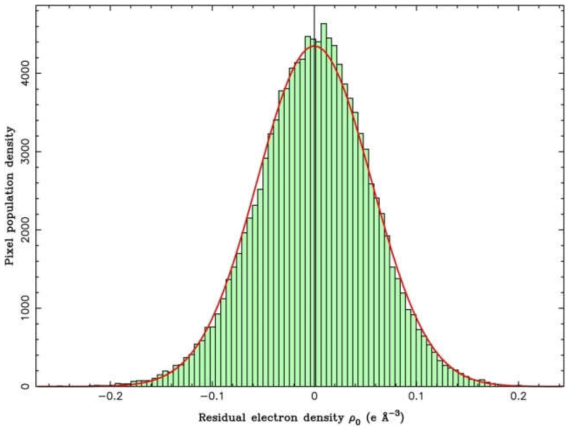

Figure S3 Fractal dimension plot for data (sinθ/λ≤0.8$Å^{-1}$)

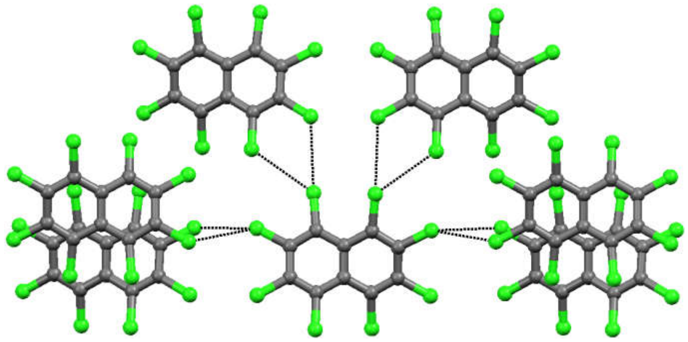

Figure S4 Cl•••Cl Interaction motifs of top half of OCN

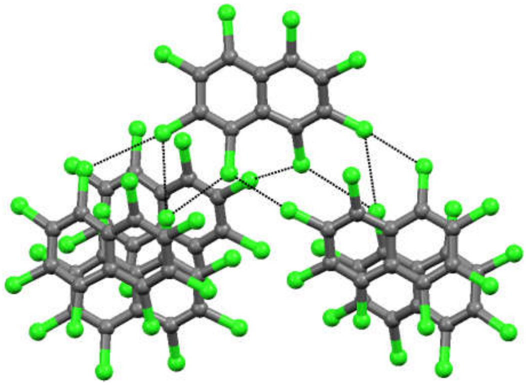

Figure S5 Cl•••Cl Interaction motifs of bottom half of OCN

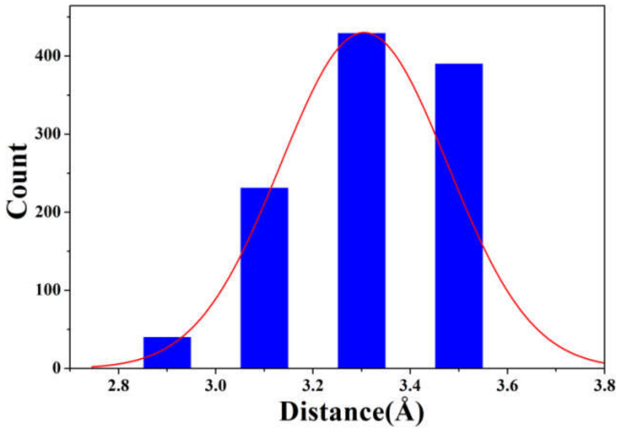

Figure S6 Histogram showing a total of 421 hits having intramolecular Cl•••Cl interactions

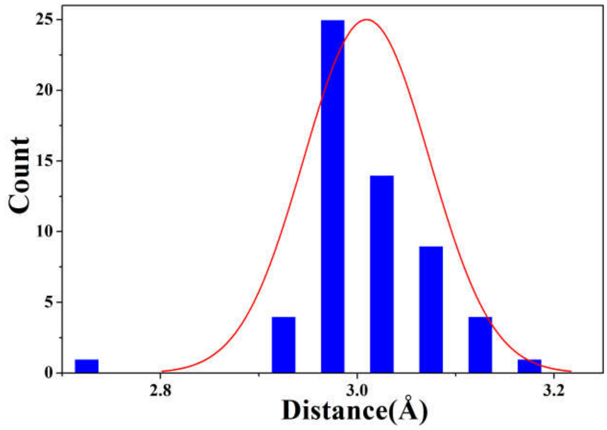

Figure S7 Histogram of *peri* Cl•••Cl distance showing maximum case around 3±0.025Å

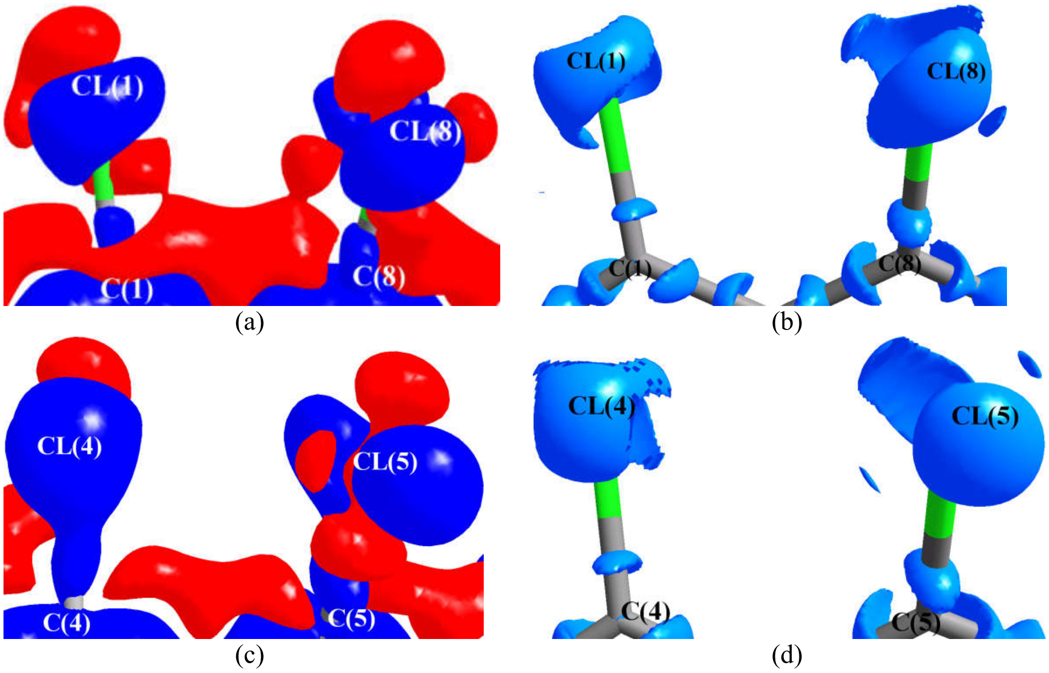

Figure S8 3D deformation density; (b) 3D Laplacian plot of the Cl(1)•••Cl(8) interaction region; (c) 3D deformation density and (d) 3D Laplacian plot of the Cl(4)•••Cl(5) interaction region. Blue represents charge concentration (CC) and red represents charge depletion (CD) in deformation maps drawn at the intervals of ±0.08 eÅ^{-3}. 3D Laplacian isosurfaces is plotted at -17.5eÅ^{-5}.

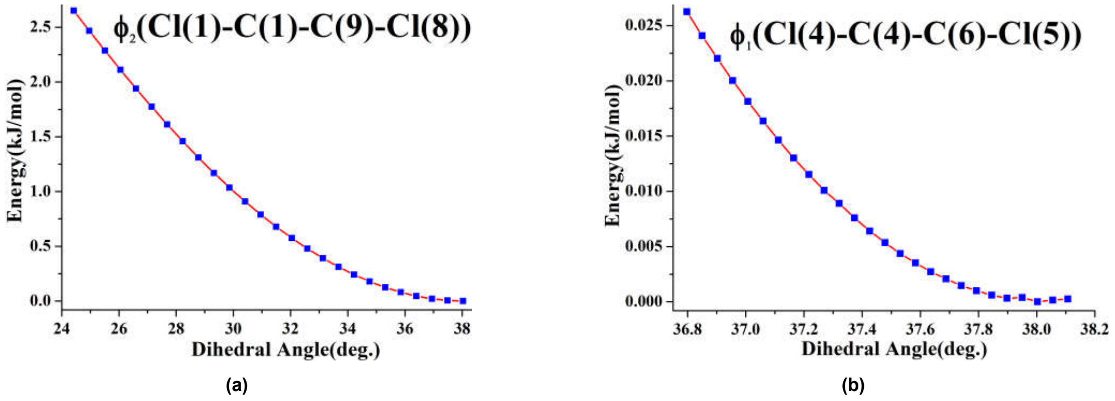

Figure S9 (a) Potential energy scan for dihedral angle ϕ_2 (b) Potential energy scan for dihedral angle ϕ_1

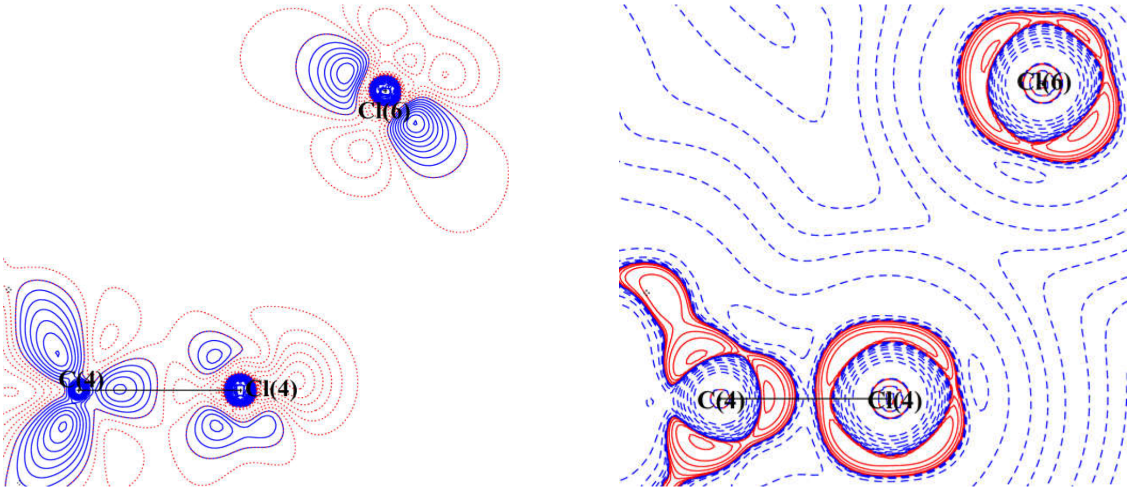

(a) (b)

Figure S10 (a) 2D deformation density; (b) 2D Laplacian plot of the intermolecular Cl(4)•••Cl(6) interaction region. Blue (solid lines) and red (broken lines) colors represent positive, negative contours respectively (reversed in case of Laplacian).Contours are drawn at the intervals of ±0.05 e Å^{-3}. Laplacian is plotted on logarithmic contours.

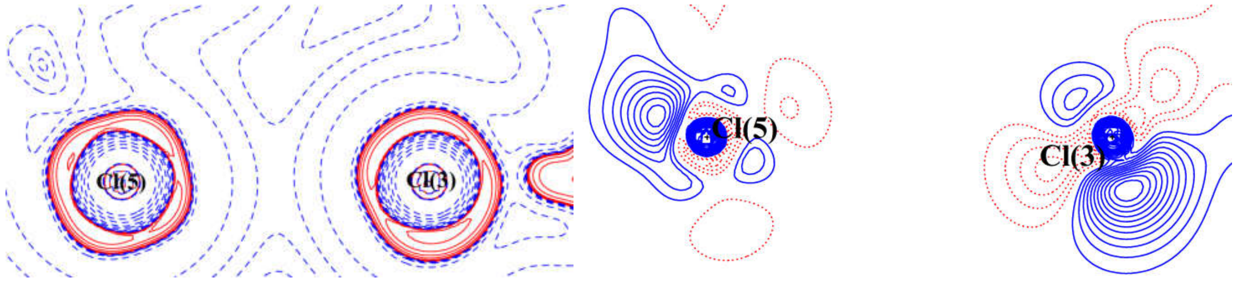

(a) (b)

Figure S11 (a) 2D deformation density; (b) 2D Laplacian plot of the intermolecular Cl(5)•••Cl(3) interaction region. Blue (solid lines) and red (broken lines) colors represent positive, negative contours respectively (reversed in case of Laplacian).Contours are drawn at the intervals of ±0.05 e Å^{-3}. Laplacian is plotted on logarithmic contours.

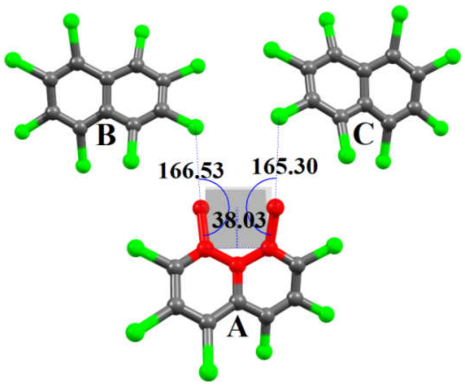

Figure S12 Molecular conformations showing the angular geometries of the **type II** Cl•••Cl interactions in the geometry of trimer **T1**

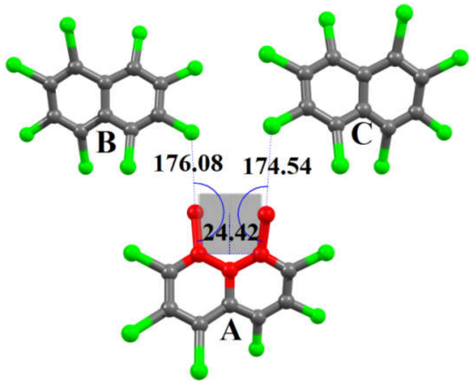

Figure S13 Molecular conformations showing the angular geometries of the **type II** Cl•••Cl interactions in the crystal geometry of trimer **T1°**

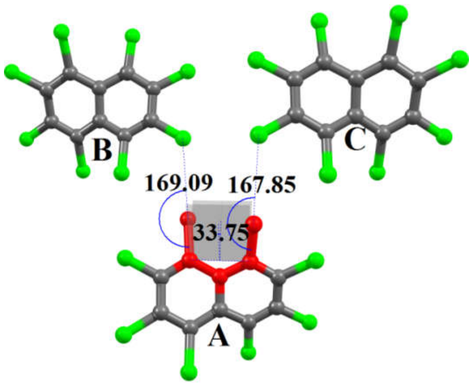

Figure S14 Molecular conformations showing the angular geometries of the **type II** Cl•••Cl interactions in the optimized geometry of trimer **T1′**.

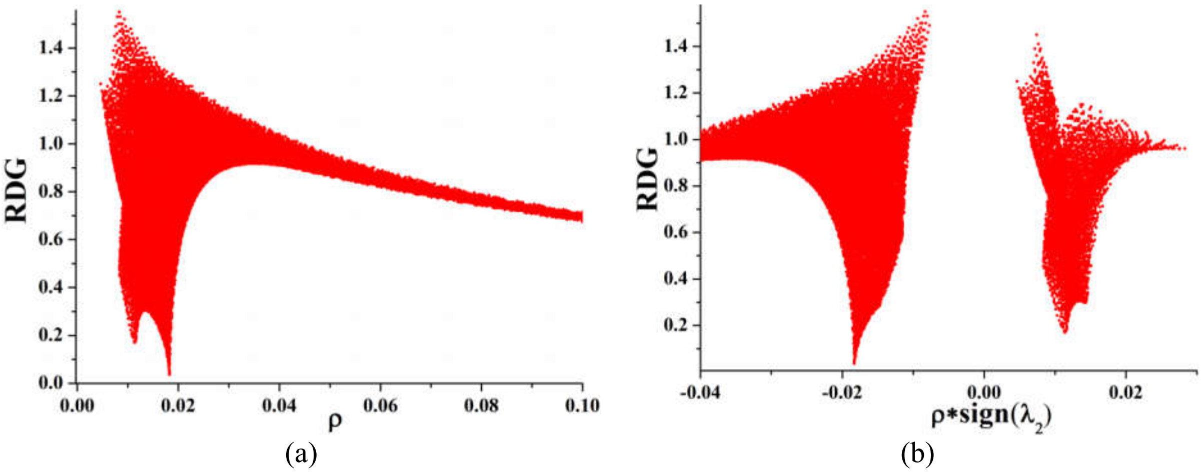

Figure S15 (a) Plot of RDG (reduced density gradient) vs. electron density (ρ). (b) Plot of RDG (reduced density gradient) vs. electron density electron density multiplied by the sign of the second Hessian eigen value (ρ*sign(λ_2)) for *peri* interaction between Cl(1)•••Cl(8).

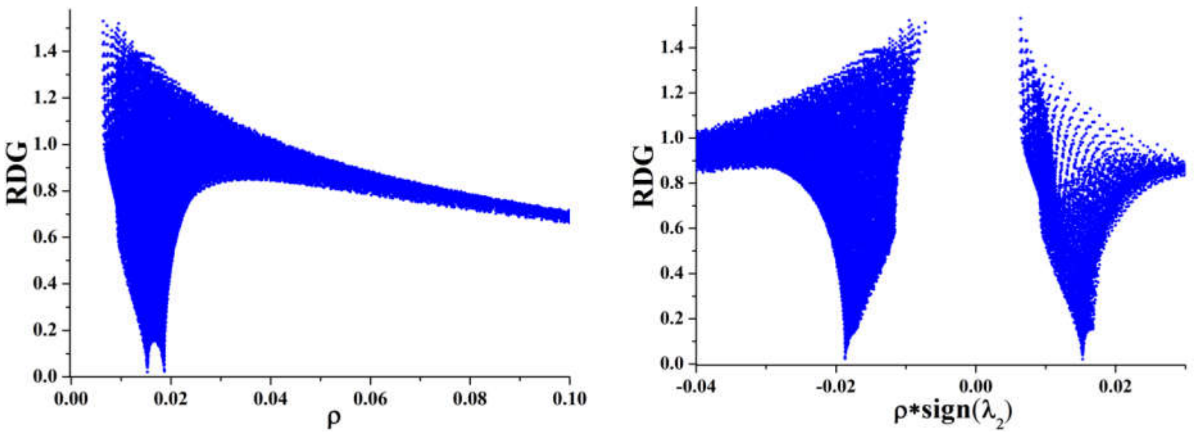

(a) (b)

Figure S16 (a) Plot of RDG (reduced density gradient) vs. electron density (ρ). (b) Plot of RDG (reduced density gradient) vs. electron density electron density multiplied by the sign of the second Hessian eigen value (ρ*sign(λ2)) for *peri* interaction between Cl(4)•••Cl(5).

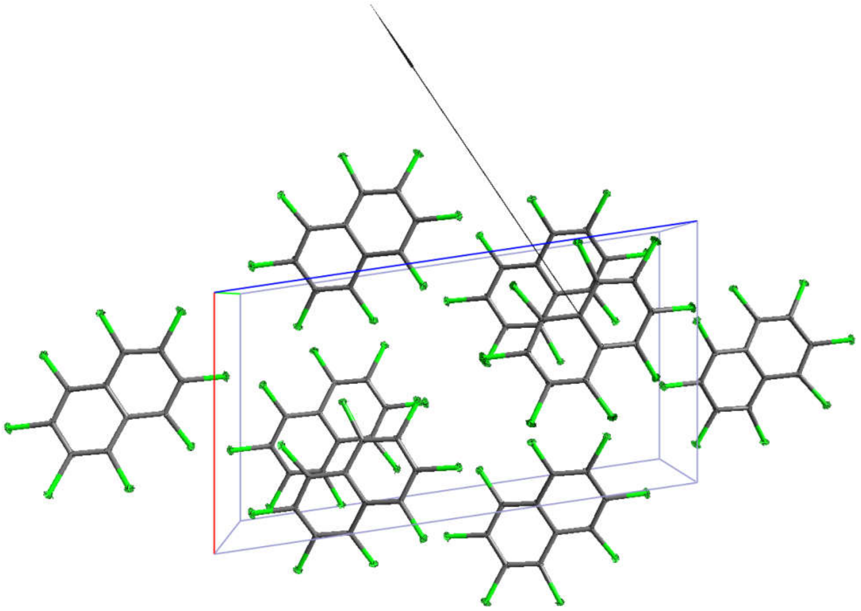

Figure S17 Molecular packing diagram of Octacholoronaphthalene (OCN) viewed along b axis. The right arrow indicates the direction of dipole moment vector. Blue indicates the c axis, red indicates the a axis, green indicates the b axis.

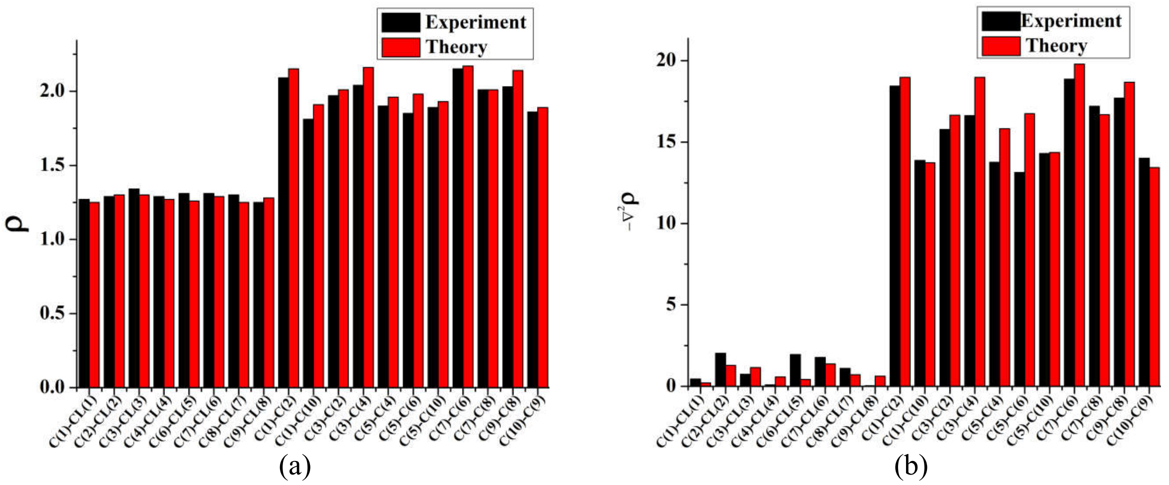

Figure S18 (a) Comparison of ρ values for all covalent bonds obtained from experiment and theory. (b) Comparison of $\nabla^2\rho$ values for all covalent bonds obtained from experiment and theory.

Table S1 Monopole Populations, Radial Parameters and Net Atomic Charges.

Atom	P_{val}	Kappa	P00	Kappa'	Atomic charge
CL(1)	6.939	1.023	0	0.994	0.061
CL(2)	6.797	1.024	0	0.985	0.203
CL(3)	7.166	1.017	0	1.022	-0.166
CL(4)	7.213	1.018	0	0.981	-0.214
CL(5)	7.104	1.022	0	0.989	-0.104
CL(6)	7.105	1.021	0	0.976	-0.105
CL(7)	6.873	1.023	0	1.009	0.127
CL(8)	6.940	1.020	0	0.990	0.060
C(1)	3.860	1.024	0	0.976	0.140
C(2)	4.018	1.020	0	0.974	-0.018
C(3)	3.981	1.015	0	0.994	0.019
C(4)	4.195	1.007	0	1.014	-0.195
C(10)	3.811	1.038	0	0.988	0.190
C(5)	4.215	1.003	0	1.038	-0.215
C(6)	4.016	1.020	0	0.994	-0.016
C(7)	4.069	1.007	0	0.980	-0.069
C(8)	3.792	1.034	0	0.992	0.208

C(9)	3.906	1.023	0	0.967	0.094

Table S2 Dipole Population Parameters.

Atom	D11+	D11-	D10	Kappa'
CL(1)	0.077	-0.024	-0.014	0.994
CL(2)	-0.006	0.016	-0.004	0.985
CL(3)	-0.075	-0.052	0.007	1.022
CL(4)	0.068	0.002	0.042	0.981
CL(5)	0.049	0.034	0.007	0.989
CL(6)	-0.036	-0.038	-0.034	0.976
CL(7)	-0.010	0.011	0.025	1.009
CL(8)	0.033	-0.024	-0.003	0.990
C(1)	-0.013	0.012	0.027	0.976
C(2)	-0.003	0.059	0.023	0.974
C(3)	-0.015	0.018	0.021	0.994
C(4)	-0.021	0.010	0.027	1.014
C(10)	-0.013	0.008	-0.002	0.988
C(5)	-0.018	0.020	0.022	1.038
C(6)	-0.009	0.022	0.036	0.994
C(7)	-0.019	0.022	0.040	0.980
C(8)	0.012	0.054	-0.021	0.992
C(9)	-0.014	-0.038	-0.027	0.967

Table S3 Quadrupole Population Parameters.

Atom	Q20	Q21+	Q21-	Q22+	Q22-	Kappa'
CL(1)	-0.222	0.075	0.135	0.180	-0.010	0.994
CL(2)	-0.325	0.019	-0.029	0.121	0.059	0.985
CL(3)	-0.197	-0.116	-0.081	0.199	0.062	1.022
CL(4)	-0.229	0.113	-0.083	0.221	-0.070	0.981

CL(5)	-0.186	0.158	0.048	0.165	-0.096	0.989
CL(6)	-0.354	-0.140	-0.018	0.129	0.075	0.976
CL(7)	-0.223	-0.049	-0.069	0.189	0.069	1.009
CL(8)	-0.201	0.064	-0.015	0.217	-0.016	0.990
C(1)	0.053	-0.011	-0.019	-0.002	-0.046	0.976
C(2)	0.054	-0.026	-0.045	-0.028	0.009	0.974
C(3)	0.025	-0.004	-0.013	-0.022	0.024	0.994
C(4)	0.032	-0.003	-0.056	-0.030	-0.030	1.014
C(10)	0.005	-0.030	-0.004	-0.042	0.004	0.988
C(5)	0.038	0.014	-0.028	-0.005	-0.023	1.038
C(6)	0.055	0.000	-0.035	-0.019	0.021	0.994
C(7)	0.078	-0.025	-0.063	-0.026	-0.011	0.980
C(8)	0.007	-0.020	-0.032	-0.011	-0.022	0.992
C(9)	0.026	-0.002	0.003	-0.045	-0.004	0.967

Table S4 Octupole Population Parameters.

Atom	O30	O31+	O31-	O32+	O32-	O33+	O33-	Kappa'
CL(1)	0.069	0.010	0.007	-0.044	0.005	-0.020	0.018	0.994
CL(2)	0.033	0.018	-0.038	0.010	-0.009	0.022	0.008	0.985
CL(3)	0.031	0.011	0.023	0.012	0.034	0.085	-0.015	1.022
CL(4)	0.025	0.040	-0.001	0.034	0.055	-0.001	0.028	0.981
CL(5)	0.041	0.038	0.005	-0.025	0.056	-0.036	0.024	0.989
CL(6)	0.048	-0.023	0.029	0.044	0.045	0.077	0.005	0.976
CL(7)	0.041	0.000	0.055	0.038	-0.013	0.002	0.016	1.009
CL(8)	0.054	0.008	0.033	-0.019	0.030	-0.012	-0.011	0.990
C(1)	0.215	-0.001	-0.020	0.205	0.047	0.026	0.032	0.976
C(2)	0.244	-0.013	0.020	0.189	0.036	-0.017	0.000	0.974
C(3)	0.170	-0.025	0.016	0.184	-0.019	0.006	0.024	0.994
C(4)	0.211	-0.010	-0.006	0.163	0.035	0.006	-0.001	1.014

C(5)	0.219	0.018	0.030	0.145	-0.014	0.016	0.016	0.988
C(6)	0.205	-0.020	0.011	0.147	0.015	-0.038	0.007	1.038
C(7)	0.216	0.003	0.009	0.194	-0.035	0.005	0.004	0.994
C(8)	0.230	-0.013	0.002	0.208	0.028	0.022	0.023	0.980
C(9)	0.213	-0.005	0.008	0.178	0.038	-0.039	0.006	0.992
C(10)	0.251	-0.066	0.020	0.195	0.001	0.008	0.037	0.967

Table S5 Hexadecapole Population Parameters

Atom	H40	H41+	H41-	H42+	H42-	H43+	H43-	H44+	H44-	Kappa'
CL(1)	-0.008	0.009	0.03	-0.077	0.01	0.044	0.03	0.01	0.013	0.994
CL(2)	0.129	-0.011	0.059	-0.039	0.01	0.029	-0.026	0.032	0.054	0.985
CL(3)	0.02	0.004	-0.025	-0.011	-0.033	-0.006	-0.048	0.006	-0.042	1.022
CL(4)	-0.033	0.007	-0.036	-0.022	0.032	0.026	-0.029	0.032	-0.011	0.981
CL(5)	-0.008	-0.002	0.016	-0.051	0.025	0.064	0.013	0.021	-0.031	0.989
CL(6)	0.118	0.06	0	0.043	-0.025	-0.026	-0.017	0.023	-0.018	0.976
CL(7)	0.013	0.008	-0.057	-0.017	-0.02	0.013	-0.043	0.073	0.039	1.009
CL(8)	-0.011	0.009	-0.052	-0.039	0.007	0.057	-0.055	0.024	0.013	0.990
C(1)	0	0	0	0	0	0	0	0	0	0.976
C(2)	0	0	0	0	0	0	0	0	0	0.974
C(3)	0	0	0	0	0	0	0	0	0	0.994
C(4)	0	0	0	0	0	0	0	0	0	1.014
C(10)	0	0	0	0	0	0	0	0	0	0.988
C(5)	0	0	0	0	0	0	0	0	0	1.038
C(6)	0	0	0	0	0	0	0	0	0	0.994
C(7)	0	0	0	0	0	0	0	0	0	0.98
C(8)	0	0	0	0	0	0	0	0	0	0.992
C(9)	0	0	0	0	0	0	0	0	0	0.967

Table S6 Aim charge of the individual atoms derived from the experimental (Q_{exp}) and theoretical (Q_{theo}) multipole modeled electron density.

Atom	Q_{exp}	Q_{theory}
CL(1)	-0.0369	-0.1818
CL(2)	**0.1108**	**-0.1351**
CL(3)	-0.2626	-0.1513
CL(4)	**-0.3238**	**-0.0740**
CL(5)	-0.2123	-0.0333
CL(6)	-0.1954	-0.1388
CL(7)	0.0121	-0.0455
CL(8)	-0.0258	-0.1698
C(1)	0.2185	0.0000
C(2)	0.0715	0.0209
C(3)	0.1256	0.0956
C(4)	-0.0633	0.1798
C(10)	0.1457	0.1916
C(5)	-0.0856	0.2308
C(6)	0.0506	0.0881
C(7)	0.0707	0.0543
C(8)	0.2551	-0.0240
C(9)	0.1471	0.1006

Table S7 Electrostatic Potential (eÅ^{-1}) calculated at the nuclear sites of each atom. V_{Exp}-Potential calculated from the experimental multipole model; V_{Theory}-Potential calculated from the theoretical multipole model.

Atom	V_{Exp}	V_{Theory}
CL(1)	-121.666	-121.853
CL(2)	**-121.610**	**-121.764**
CL(3)	-122.066	-121.710

CL(4)	**-122.210**	**-121.555**
CL(5)	-122.135	-121.493
CL(6)	-122.032	-121.645
CL(7)	-121.691	-121.645
CL(8)	-121.630	-121.825
C(1)	-27.556	-27.828
C(2)	-27.713	-27.771
C(3)	-27.912	-27.721
C(4)	-28.043	-27.587
C(10)	-27.911	-27.595
C(5)	-28.039	-27.490
C(6)	-27.930	-27.644
C(7)	-27.688	-27.695
C(8)	-27.576	-27.806
C(9)	-27.636	-27.769

Table S8 Topological features obtained for all covalent bonds in OCN. r_1 and r_2 are the distances from the BCP to the first atom (A) and second atom (B), respectively. The interaction length, $R_{ij} = (r_1 + r_2)$. The values obtained from periodic calculations using the M062X/TZVP method are given in italics

Atom A	Atom B	R_{ij}(Å) (A-B)	r_1(Å) (A-CP)	r_2(Å) (CP-B)	$\rho(r)_{cp}$ (eÅ^{-3})	$\nabla^2\rho$ (eÅ^{-5})	λ_1	λ_2	λ_3	ε
C(1)	CL(1)	1.7211	0.7968	0.9243	1.27	-0.45	-7.24	-6.78	13.57	0.07
		1.7207	*0.8038*	*0.9169*	*1.25*	*-0.22*	*-6.69*	*-6.14*	*12.61*	*0.09*
C(2)	CL(2)	1.7119	0.7868	0.9251	1.29	-2.03	-8.43	-6.99	13.4	0.21
		1.7116	*0.7956*	*0.916*	*1.30*	*-1.29*	*-7.32*	*-6.32*	*12.36*	*0.16*
C(3)	CL(3)	1.7131	0.7875	0.9256	1.34	-0.74	-7.93	-6.53	13.72	0.21
		1.7130	*0.7931*	*0.9199*	*1.3*	*-1.15*	*-7.07*	*-6.61*	*12.52*	*0.07*
C(4)	CL(4)	1.7220	0.7981	0.9239	1.29	-0.09	-7.46	-6.47	13.84	0.15
		1.7193	*0.8007*	*0.9186*	*1.27*	*-0.58*	*-7.30*	*-5.59*	*12.31*	*0.31*
C(6)	CL(5)	1.7203	0.7927	0.9276	1.31	-1.95	-7.34	-6.48	11.87	0.13

		1.7201	*0.8027*	*0.9174*	*1.26*	*-0.43*	*-7.14*	*-6.31*	*13.02*	*0.13*
C(7)	CL(6)	1.7112	0.7911	0.9201	1.31	-1.78	-8.96	-6.53	13.71	0.37
		1.7111	*0.7917*	*0.9194*	*1.29*	*-1.38*	*-7.13*	*-6.42*	*12.18*	*0.11*
C(8)	CL(7)	1.7132	0.7912	0.922	1.30	-1.1	-7.93	-6.26	13.09	0.27
		1.7129	*0.7958*	*0.9171*	*1.25*	*-0.71*	*-6.96*	*-5.93*	*12.18*	*0.17*
C(9)	CL(8)	1.7212	0.7947	0.9265	1.25	-0.04	-7.44	-6.11	13.51	0.22
		1.7204	*0.8114*	*0.909*	*1.28*	*-0.63*	*-6.77*	*-6.24*	*12.38*	*0.08*
C(1)	C(2)	1.3841	0.6831	0.701	2.09	-18.44	-18.14	-12.1	11.79	0.50
		1.3841	*0.6800*	*0.7041*	*2.15*	*-18.97*	*-16.68*	*-12.78*	*10.49*	*0.30*
C(1)	C(10)	1.4352	0.7156	0.7196	1.81	-13.88	-15.24	-10.59	11.95	0.44
		1.4354	*0.7336*	*0.7018*	*1.91*	*-13.74*	*-13.96*	*-11.6*	*11.83*	*0.2*
C(3)	C(2)	1.4188	0.6903	0.7285	1.97	-15.77	-16.07	-11.75	12.05	0.37
		1.4185	*0.6948*	*0.7237*	*2.01*	*-16.65*	*-14.88*	*-12.52*	*10.75*	*0.19*
C(3)	C(4)	1.3797	0.6707	0.709	2.04	-16.63	-16.86	-11.4	11.63	0.48
		1.3799	*0.6951*	*0.6848*	*2.16*	*-18.98*	*-16.95*	*-13.13*	*11.09*	*0.29*
C(5)	C(4)	1.4330	0.7001	0.7329	1.9	-13.76	-15.59	-11.17	13.00	0.40
		1.4333	*0.7059*	*0.7274*	*1.96*	*-15.82*	*-14.85*	*-12.63*	*11.66*	*0.18*
C(5)	C(6)	1.4332	0.7018	0.7314	1.85	-13.14	-14.73	-11.03	12.62	0.34
		1.4335	*0.706*	*0.7275*	*1.98*	*-16.75*	*-14.98*	*-13.09*	*11.32*	*0.14*
C(5)	C(10)	1.4383	0.7062	0.7321	1.89	-14.3	-15.34	-11.61	12.65	0.32
		1.4381	*0.7168*	*0.7213*	*1.93*	*-14.36*	*-13.87*	*-12.28*	*11.79*	*0.13*
C(7)	C(6)	1.3804	0.6894	0.691	2.15	-18.86	-18.23	-12.62	11.99	0.44
		1.3804	*0.6951*	*0.6853*	*2.17*	*-19.79*	*-17.31*	*-13.09*	*10.6*	*0.32*
C(7)	C(8)	1.4176	0.7085	0.7091	2.01	-17.2	-16.84	-12.27	11.91	0.37
		1.4169	*0.6981*	*0.7188*	*2.01*	*-16.69*	*-15*	*-12.41*	*10.73*	*0.21*
C(9)	C(8)	1.3840	0.6586	0.7254	2.03	-17.7	-17.59	-11.63	11.51	0.51
		1.3842	*0.6879*	*0.6963*	*2.14*	*-18.68*	*-16.33*	*-12.78*	*10.44*	*0.28*
C(10)	C(9)	1.4369	0.7033	0.7336	1.86	-14.01	-15.58	-10.93	12.49	0.42
		1.4372	*0.7096*	*0.7276*	*1.89*	*-13.44*	*-13.73*	*-11.38*	*11.67*	*0.21*

References:

Chai, J.-D. & Head-Gordon, M. (2008). *Phys. Chem. Chem. Phys.* **10**, 6615-6620.

Cheeseman, J. R., Trucks, G. W., Keith, T. A. & Frisch, M. J. (1996). *J. Chem. Phys.* **104**, 5497-5509.

Dovesi, R., Saunders, V., Roetti, C., Orlando, R., Zicovich-Wilson, C., Pascale, F., Civalleri, B., Doll, K., Harrison, N. & Bush, I. (2013). *Università di Torino, Torino*

Frisch, M., Trucks, G., Schlegel, H. B., Scuseria, G., Robb, M., Cheeseman, J., Scalmani, G., Barone, V., Mennucci, B. & Petersson, G. (2009). *Inc., Wallingford, CT* **200**,

Hirshfeld, F. (1976). *Acta Crystallogr. Sect. A: Found. Crystallogr.* **32**, 239-244.

Hübschle, C. B. & Dittrich, B. (2011). *J. Appl. Crystallogr.* **44**, 238-240.

London, F. (1937). *J. phys. radium* **8**, 397-409.

Meindl, K. & Henn, J. (2008). *Acta Crystallogr. Sect. A: Found. Crystallogr.* **64**, 404-418.

Peintinger, M. F., Oliveira, D. V. & Bredow, T. (2013). *Journal of computational chemistry* **34**, 451-459.

Saleh, G., Lo Presti, L., Gatti, C. & Ceresoli, D. (2013). *J. Appl. Crystallogr.* **46**, 1513-1517.

Schäfer, A., Horn, H. & Ahlrichs, R. (1992). *J. Chem. Phys.* **97**, 2571-2577.

Tomasi, J., Mennucci, B. & Cammi, R. (2005). *Chem. Rev.* **105**, 2999-3094.

Zhao, Y. & Truhlar, D. G. (2008). *Theor. Chem. Acc.* **120**, 215-241.
